# Cinnamaldehyde and allopurinol reduce fructose-induced cardiac inflammation and fibrosis by attenuating CD36-mediated TLR4/6-IRAK4/1 signaling to suppress NLRP3 inflammasome activation

**DOI:** 10.1038/srep27460

**Published:** 2016-06-08

**Authors:** Lin-Lin Kang, Dong-Mei Zhang, Chun-Hua Ma, Jian-Hua Zhang, Ke-Ke Jia, Jia-Hui Liu, Rong Wang, Ling-Dong Kong

**Affiliations:** 1 State Key Laboratory of Pharmaceutical Biotechnology, School of Life Science, Nanjing University, Nanjing, People’s Republic of China

## Abstract

Fructose consumption induces metabolic syndrome to increase cardiovascular disease risk. Cinnamaldehyde and allopurinol possess anti-oxidative and anti-inflammatory activity to relieve heart injury in metabolic syndrome. But the mechanisms of fructose-induced cardiac injury, and cardioprotective effects of cinnamaldehyde and allopurinol are not completely understood. In this study, fructose-fed rats displayed metabolic syndrome with elevated serum ox-LDL, cardiac oxidative stress, inflammation and fibrosis. Scavenger receptor CD36, Toll-like receptor 4 (TLR4), TLR6, IL-1R-associated kinase 4/1 (IRAK4/1), nucleotide-binding domain (NOD)-like receptor protein 3 (NLRP3) inflammasome, interleukin-1β, transforming growth factor-β (TGF-β), drosophila mothers against DPP homolog (Smad) 2/3 phosphorylation and Smad4 were increased in animal and H9c2 cell models. These pathological processes were further evaluated in ox-LDL or fructose-exposed H9c2 cells pretreated with ROS scavenger and CD36 specific inhibitor, or IRAK1/4 inhibitor, and transfected with *CD36*, *NLRP3*, or *IRAK4*/*1* siRNA, demonstrating that NLPR3 inflammasome activation through CD36-mediated TLR4/6-IRAK4/1 signaling may promote cardiac inflammation and fibrosis. Cinnamaldehyde and allopurinol reduced cardiac oxidative stress to suppress NLPR3 inflammasome activation and TGF-β/Smads signaling by inhibiting CD36-mediated TLR4/6-IRAK4/1 signaling under fructose induction. These results suggest that the blockage of CD36-mediated TLR4/6-IRAK4/1 signaling to suppress NLRP3 inflammasome activation by cinnamaldehyde and allopurinol may protect against fructose-induced cardiac inflammation and fibrosis.

More evidence suggests that excess fructose consumption induces oxidative stress and inflammation to increase the incidence of metabolic syndrome, and consequently elevates the risk of heart disease^1^,^2^,^3^,^4^,^5^. Low density lipoprotein (LDL) oxidation under oxidative stress to form oxidized LDL (ox-LDL) is a main step in the development of cardiovascular disease, closely linking to cardiac structural and functional damage with inflammation response^6^. Fructose consumption can induce high levels of serum LDL and ox-LDL in adult or children subjects^2^,^7^. The scavenger receptor CD36 mediates recognition and uptake of ox-LDL. Fructose feeding also increases cardiac CD36 protein levels in the basal and insulin-stimulated states in rats^8^. The interaction between CD36 and ox-LDL induces the secretion of inflammatory cytokine interleukin (IL)-1β^9^,^10^,^11^, which is mediated by assembly of the activation of nucleotide-binding domain (NOD)-like receptor protein 3 (NLRP3) inflammasome^12^. Targeting CD36^−/−^ blocks NLRP3 inflammasome activation and antagonizes IL-1β secretion *in vivo*^13^. Thioredoxin-interacting protein (TXNIP) inhibits thioredoxin to increase reactive oxygen species (ROS) production in cells^14^. Cardiac TXNIP up-regulation is observed in mice with cardiomyocyte impairment of streptozotocin-induced diabetes^15^ and myocardial ischemia reperfusion injury^16^. Of note, CD36 inhibitor sulfosuccinimidyl oleate sodium (SSO) blocks ceramide-induced TXNIP over-expression in rat insulinoma cell line INS-1^17^. ROS scavenger dissociates TXNIP from NLRP3, and inhibits NLRP3 inflammasome activation in cardiac microvascular endothelial of C57BL/6J mice with simulated ischemia/reperfusion injury^18^. Transforming growth factor-β (TGF-β) is a profibrosis factor in fibrosis disease. Cardiac NLRP3 inflammasome activation participates in myocardial fibrosis, which is related to IL-1β-induced inflammation and high TGF-β1 level in the transverse aortic constriction-induced mouse left ventricular remodeling^19^. NLRP3 and IL-1β mRNA levels are increased in myocardial fibroblasts post-myocardial infarction of rodents^20^. NLRP3-deficient cardiac fibroblast displays impaired differentiation and R-drosophila mothers against DPP homolog (Smad) activation in response to TGF-β^21^. Oxidative stress and high TGF-β1 level are observed in fructose-induced rat cardiac fibrosis^22^. However, the molecular basis of cardiac CD36-mediated NLRP3 inflammasome activation and fibrosis under fructose-induced oxidative stress is still poorly defined.

Toll-like receptors (TLRs) are a key link between innate immunity and cardiovascular diseases^23^. CD36-regulated ox-LDL triggers inflammatory signaling through assembly of heterodimer of TLR4 and TLR6^24^. Myeloid differentiation primary response gene 88 associates with cytoplasmic portion of TLRs and then recruits IL-1R-associated kinase 1 (IRAK1) by IRAK4^25^,^26^. IRAK4 knockout mice are completely nonresponsive to TLR signaling^27^. Deletion of IRAK4 or IRAK1 leads to defective NLRP3 inflammasome activation^28^,^29^. Therefore, TLR4/6-IRAK4/1 pathway may be required for CD36-mediated NLRP3 inflammasome activation in fructose-induced cardiac injury.

Cinnamaldehyde is a key flavor constituent isolated from the bark of *Cinnamonum cassia* Presl, which is commonly used as Chinese medicine for gastritis, dyspepsia, blood circulation disturbance and inflammation^30^. Cinnamaldehyde decreases serum levels of total triglyceride (TG) and total cholesterol (TC) in mice and patients with diabetes^31^,^32^. It reduces ROS production and IL-1β secretion to alleviate metabolic disturbance-associated inflammation in murine RAW 264.7 or J774A.1 macrophages, suppresses plasma TLR4 expression and inflammatory cell infiltrate in myocardium from viral myocarditis mice^33^,^34^,^35^. Cinnamaldehyde with anti-oxidative and anti-inflammatory property also alleviates ischemic myocardial injury of rats^36^. Allopurinol, a xanthine oxidase (XOD) inhibitor, decreases serum ox-LDL concentrations in patients with gout, reduces 24-h daytime systolic blood pressure (SBP) and low density lipoprotein cholesterol (LDL-c) levels in healthy adult men with excessive fructose intake^37^,^38^. In our previous studies, allopurinol ameliorated fructose-induced metabolic syndrome and protects tissue injury by inhibiting NLRP3 inflammasome activation and IL-1β production^39^,^40^. Recently, allopurinol is found to restore a high-fat and high-fructose diet-induced cardiomyocyte oxidative stress, inflammation and hypertrophy in mice^41^, and alleviates cardiac ischemia in insulin resistance through inhibiting low grade inflammation and angiotensin system in rats fed with a high fructose and fat diet^42^. Thus, the cardioprotective effects of cinnamaldehyde and allopurinol against cardiac inflammation may be involved in heart injury under fructose-induced oxidative stress, but the molecular mechanism has not been understood yet.

Therefore, we hypothesized that cinnamaldehyde and allopurinol may reduce oxidative stress to inhibit NLRP3 imflammasome activation via CD36-meidated TLR4/6-IRAK4/1-dependent manner in the pathogenesis of fructose-induced cardiac injury. To investigate our hypothesis, we constructed fructose feeding-induced rat model with high serum ox-LDL level, cardiac oxidative stress, inflammation and fibrosis in metabolic syndrome, and evaluated protective effects of cinnamaldehyde and allopurinol in this animal model. We also investigated the mechanisms of cinnamaldehyde and allopurinol on the reduction of cardiac inflammation and fibrosis in rat myocardial cell line H9c2 cells pretreated with ROS scavenger N-acetylcysteine (NAC), SSO or IRAK1/4 inhibitor I, or transfected with *CD36*, *NLRP3*, *IRAK4* or *IRAK1* siRNA under fructose induction. This study suggests that cinnamaldehyde and allopurinol may protect against fructose-induced cardiac inflammation and fibrosis associated with metabolic syndrome.

## Results

### Cinnamaldehyde and allopurinol alleviate fructose-induced metabolic syndrome in rats

**Figure 1:**
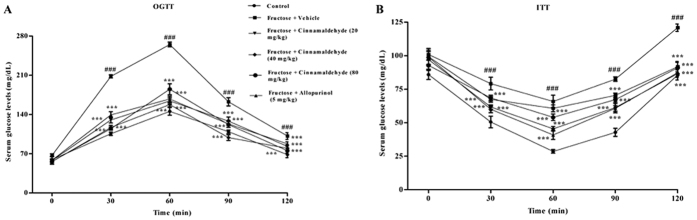
Cinnamaldehyde and allopurinol improve insulin resistance in fructose-fed rats. Plasma glucose profiles in OGTT (**A**) and ITT (**B**) assays were measured (n = 10). Data are expressed as the mean ± SEM. ^###^*P* < 0.001 *vs* normal animal control group; ***P < 0.001 *vs* fructose-vehicle animal group.

Consistent with our previous work^43^, fructose-fed rats developed insulin resistance assayed by the results of oral glucose tolerance test (OGTT) and insulin tolerance test (ITT) (Fig. 1A,B). Body weight was significantly increased, while 24 h-food intake was decreased in this animal model (Table 1). Systolic blood pressure (SBP), serum LDL-c, ox-LDL, TG and TC levels were significantly elevated in fructose-fed rats (Tables 1 and 2).

20, 40 and 80 mg/kg cinnamaldehyde was found to attenuate fructose-induced these changes in rats in a dose-dependent manner, except 20 mg/kg cinnamaldehyde had no effect on body weight (Table 1), SBP (Table 1) and ox-LDL (Table 2). Allopurinol at 5 mg/kg had similar effects in this animal model (Fig. 1, Tables 1 and 2). These data indicate that cinnamaldehyde and allopurinol improve metabolic syndrome including insulin resistance, obesity, hypertension and hyperlipidaemia in fructose-fed rats.

### Cinnamaldehyde and allopurinol reduce fructose-induced cardiac hypertrophy and fibrosis

**Figure 2:**
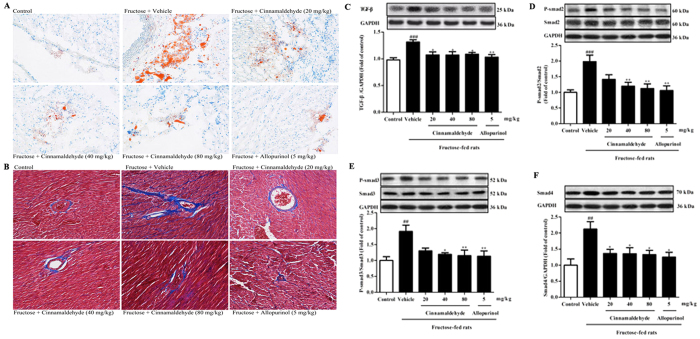
Cinnamaldehyde and allopurinol reduce fructose-induced cardiac hypertrophy and fibrosis in rats. Histology of heart sections in different groups was examined by oil red O (**A**) and Masson trichrome (**B**) staining, respectively (magnification ×200). Protein levels of cardiac TGF-β (**C**), p-Smad2/3 (**D**,**E**) and Smad4 (**F**) were measured. Relative protein levels of TGF-β and Smad4 were normalized to GAPDH (n = 7), respectively. Relative protein levels of p-Smad2/3 were normalized to Smad2/3, respectively (n = 7). Data are expressed as the mean ± SEM. ^##^*P* < 0.01, ^###^*P* < 0.001 *vs* normal animal control group; *P < 0.05, **P < 0.01 *vs* fructose-vehicle animal group.

**Figure 3:**
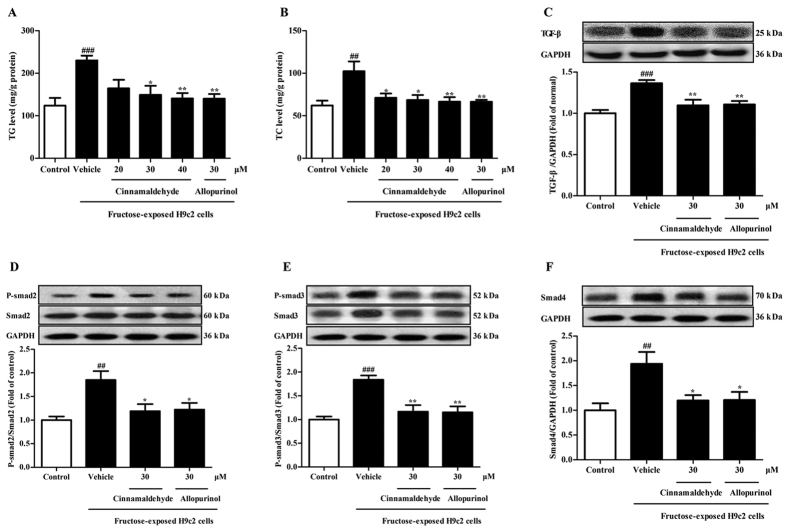
Cinnamaldehyde and allopurinol reduce cellular TG, TC, TGF-β, p-Smad2/3 and Smad4 in fructose-exposed H9c2 cells. Cellular TG (**A**) and TC (**B**) levels were shown, respectively (n = 6). Protein levels of cellular TGF-β (**C**), p-Smad2/3 (**D**,**E**) and Smad4 (**F**) were measured. Relative protein levels of TGF-β and Smad4 were normalized to GAPDH (n = 7). Relative protein levels of p-Smad2/3 were normalized to Smad2/3, respectively (n = 7). Data are expressed as the mean ± SEM. ^##^*P* < 0.01, ^###^*P* < 0.001 *vs* normal cell control group; *P < 0.05, **P < 0.01 *vs* fructose-vehicle cell group.

Furthermore, heart-to-body weight (HW/BW) was elevated significantly in fructose-fed rats (Table 1). Oil red O staining showed heart lipid accumulation (Fig. 2A) obviously in fructose-fed rats with high heart TG and TC levels (Table 2). Particularly, extensive interstitial fibrosis was observed, as well as bundles of myofibers were packed less tightly and separated by thick layers of fibrous tissue in the heart of fructose-fed rats (Fig. 2B). Concomitantly, cardiac protein levels of TGF-β, and its downstream phosphorylated Smad2/3 (p-Smad2/3) and Smad4 were significantly increased (Fig. 2C–F), accompanying with high heart hydroxyproline levels (Table 2) in this animal model. To address this, H9c2 cells were incubated with 1 mM fructose for 24 h. Cellular TG and TC levels, as well as TGF-β, p-Smad2/3 and Smad4 protein levels were significantly increased in fructose-exposed H9c2 cells (Fig. 3A–F). These data further demonstrate cardiac hypertrophy and fibrosis in fructose-induced metabolic syndrome of animals^5^.

Cinnamaldehyde (40 and 80 mg/kg), as well as allopurinol (5 mg/kg) alleviated fructose-induced heart pathology and lipid accumulation in rats (Fig. 2A,B, Table 2). Cinnamaldehyde (40 and 80 mg/kg) and allopurinol (5 mg/kg) were found to decrease HW/BW (Table 1), and down-regulate cardiac TGF-β, p-Smad2/3, Smad4 and hydroxyproline levels in fructose-fed rats (Fig. 2C–F, Table 2). 20 mg/kg cinnamaldehyde only reduced TGF-β and Smad4 obviously in the heart of fructose-fed rats (Fig. 2C,F).

In the present study, cinnamaldehyde at 30 and 40 μM and allopurinol at 30 μM attenuated fructose-induced elevation of TG and TC levels in H9c2 cells (Fig. 3A,B). 20 μM cinnamaldehyde only reduced TC levels in this cell model (Fig. 3B). Cinnamaldehyde and allopurinol both at 30 μM restored fructose-induced changes of TGF-β, p-Smad2/3 and Smad4 protein levels in H9c2 cells (Fig. 3C–F). These results indicate that cinnamaldehyde and allopurinol may have the potential to protect against fructose-induced cardiac hypertrophy and fibrosis in metabolic syndrome of rats.

### Cinnamaldehyde and allopurinol reduce fructose-induced cardiac oxidative stress and ROS to down-regulate CD36

**Figure 4:**
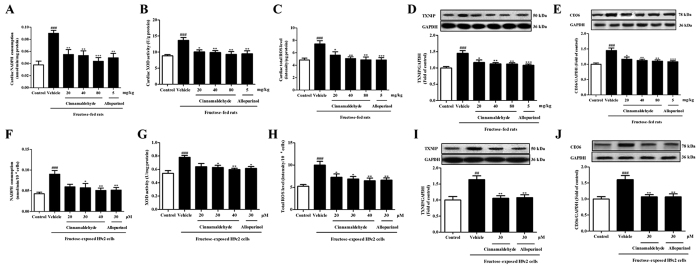
Cinnamaldehyde and allopurinol reduce oxidative stress and CD36 expression in the heart of fructose-fed rats and fructose-exposed H9c2 cells. Cardiac NADPH oxidase (**A**), XOD (**B**) and ROS (**C**) were analyzed (n = 10), respectively. Protein levels of cardiac TXNIP (**D**) and CD36 (**E**) were measured, and relative protein levels were normalized to GAPDH (n = 7). Cellular NADPH oxidase (**F**), XOD (**G**), ROS (**H**), TXNIP (**I**), and CD36 (**J**) were also determined in H9c2 cells co-incubated with fructose, cinnamaldehyde and allopurinol (n = 6), respectively. Data are expressed as the mean ± SEM. ^##^*P* < 0.01, ^###^*P* < 0.001 *vs* normal animal control group; *P < 0.05, **P < 0.01, ***P < 0.001 *vs* fructose-vehicle animal group or fructose-vehicle cell group.

Cardiac nicotinamide adenine dinucleotide phosphate (NADPH) oxidase and XOD activity were significantly increased in fructose-fed rats (Fig. 4A,B), with the increase of cardiac ROS, TXNIP (Fig. 4C,D), hydrogen peroxide (H_2_O_2_), superoxide anion (O_2_^·−^), hydroxyl radical (^·^OH) and malondialdehyde (MDA) (Table 3). Glutathione (GSH)/oxidized glutathione (GSSG) ratio (Table 3), and anti-oxidation enzyme activity of superoxidase dismutase (SOD) and catalase (CAT) (Table 3) were significantly reduced in the heart of fructose-fed rats. NADPH oxidase and XOD activity, as well as ROS production and TXNIP expression were also increased in fructose-exposed H9c2 cells (Fig. 4F–I). Moreover, CD36 protein levels were up-regulated in the heart of fructose-fed rats (Fig. 4E) and fructose-exposed H9c2 cells (Fig. 4J).

**Figure 5:**
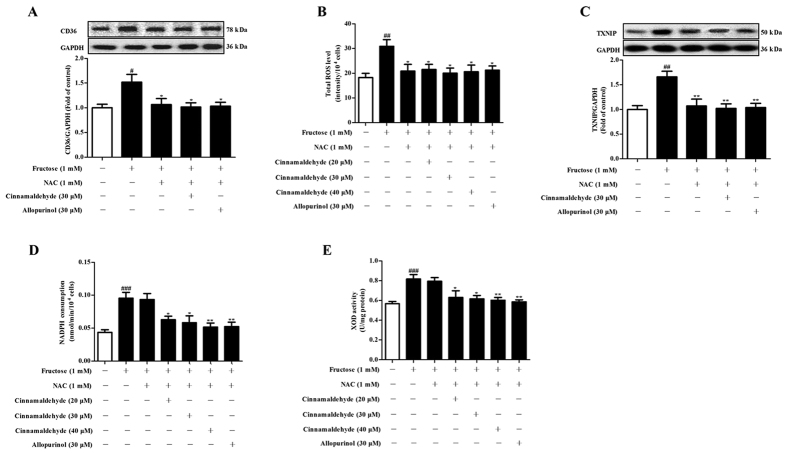
Effects of cinnamaldehyde and allopurinol on fructose-induced alteration of cellular ROS, TXNIP, NADPH oxidase, XOD and CD36 in NAC pretreated-H9c2 cells. Cellular CD36 protein levels (**A**), ROS (**B**), TXNIP protein levels (**C**), NADPH oxidase (**D**) and XOD (E) were determined in NAC-pretreated H9c2 cells co-incubated with fructose, cinnamaldehyde and allopurinol (n = 6), respectively. The relative CD36 and TXNIP protein levels were normalized to GAPDH. Data are expressed as the mean ± SEM. ^#^*P* < 0.05, ^##^*P* < 0.01, ^###^*P* < 0.001 *vs* normal cell control group; *P < 0.05, **P < 0.01 *vs* fructose-vehicle cell group, or fructose-vehicle + NAC control cell group.

**Figure 6:**
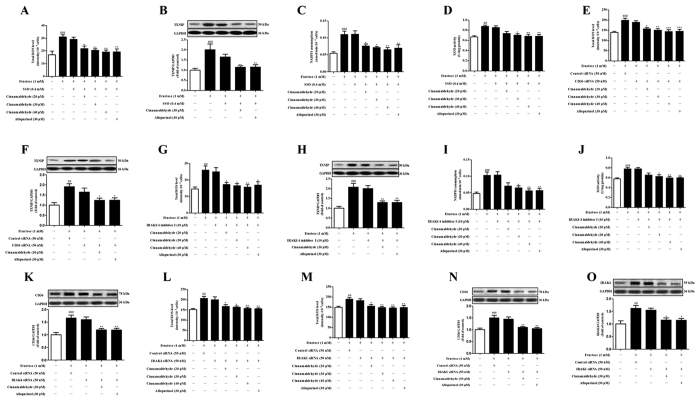
Effects of cinnamaldehyde and allopurinol on fructose-induced alteration of cellular ROS, TXNIP, NADPH oxidase, XOD and CD36 in SSO or IRAK1/4 inhibitor I pretreated-, and *CD36*, *IRAK4* or *IRAK1* siRNA transfected-H9c2 cells. Cellular ROS (**A**), TXNIP protein levels (**B**), NADPH oxidase (**C**) and XOD (**D**) were determined in SSO-pretreated H9c2 cells co-incubated with fructose, cinnamaldehyde and allopurinol (n = 6), respectively. Cellular ROS (**E**) and TXNIP protein levels (**F**) were determined in *CD36* siRNA-transfected H9c2 cells co-incubated with fructose, cinnamaldehyde and allopurinol (n = 6). Cellular ROS (**G**), TXNIP protein levels (**H**), NADPH oxidase (**I**) and XOD (**J**) were determined in IRAK1/4 inhibitor I-pretreated H9c2 cells co-incubated with fructose, cinnamaldehyde and allopurinol (n = 6), respectively. Cellular CD36 protein levels (**K**) and ROS (**L**) were determined in *IRAK4* siRNA-transfected H9c2 cells co-incubated with fructose, cinnamaldehyde and allopurinol (n = 6), respectively. Cellular ROS (**M**), CD36 (**N**) and IRAK4 (**O**) protein levels were determined in *IRAK1* siRNA-transfected H9c2 cells co-incubated with fructose, cinnamaldehyde and allopurinol (n = 6), respectively. The relative CD36, TXNIP and IRAK4 protein levels were normalized to GAPDH. Data are expressed as the mean ± SEM. ^##^*P* < 0.01, ^###^*P* < 0.001 *vs* normal cell control group; *P < 0.05, **P < 0.01, ***P < 0.001 *vs* fructose-vehicle cell group, or fructose-vehicle + SSO, IRAK1/4 inhibitor or siRNA control cell group.

To investigate whether oxidative stress contributed to cardiac CD36 up-regulation under fructose induction, H9c2 cells were pretreated with NAC (1 mM) or SSO (0.4 mM) for 0.5 or 1 h, respectively, and then co-incubated with 1 mM fructose for other 24 h. NAC prevented fructose-induced CD36 over-expression (Fig. 5A), ROS overproduction and TXNIP over-expression, but not NADPH oxidase and XOD hyperactivity (Fig. 5B–E) in H9c2 cells. SSO failed to affect fructose-induced ROS overproduction and TXNIP over-expression, as well as NADPH oxidase and XOD hyperactivity in H9c2 cells (Fig. 6A–D). In fact, ROS overproduction and TXNIP over-expression were observed in *CD36* siRNA-transfected H9c2 cells exposed to 1 mM fructose (Fig. 6E,F). Thus, fructose-driven ROS may mainly contribute to CD36 up-regulation in myocardial cells.

Ox-LDL can enhance ROS generation in endothelial cells^44^,^45^ and macrophages^46^,^47^. Fructose consumption induces high serum ox-LDL levels observed in adult or children subjects^2^,^7^, as well as in rats in the present study (Table 2). To further address above findings, H9c2 cells were exposed with ox-LDL (25 and 50 μg/mL), in the presence or absence of NAC (1 mM). Ox-LDL (25 and 50 μg/mL) induced ROS overproduction (Fig. S1A,B) and TXNIP over-expression (Fig. S1C,D) in H9c2 cells, which were attenuated by NAC. NAC and SSO also prevented ox-LDL-induced CD36 up-regulation in H9c2 cells (Fig. S2A,B). These observations further demonstrate that cardiac ROS may cause CD36 up-regulation in myocardial cells under fructose induction.

Cinnamaldehyde and allopurinol at all tested doses decreased NADPH oxidase and XOD activity (Fig. 4A,B), reduced ROS production and TXNIP expression (Fig. 4C,D), H_2_O_2_, O_2_^·−^, ·OH and MDA accumulation (Table 3), and elevated SOD and CAT activity (Table 3) in the heart of fructose-fed rats. Cinnamaldehyde at two higher doses and allopurinol also increased cardiac GSH/GSSG ratio in this animal model (Table 3). Cinnamaldehyde (30 and 40 μM) and allopurinol (30 μM) reduced NADPH oxidase and XOD activity (Fig. 4F,G) in fructose-exposed H9c2 cells. Cinnamaldehyde (20, 30 or 40 μM) and allopurinol (30 μM) significantly reduced ROS production and TXNIP expression in fructose-exposed H9c2 cells (Fig. 4H,I). Subsequently, cinnamaldehyde and allopurinol significantly down-regulated CD36 protein levels in fructose-fed rat heart (Fig. 4E) and fructose-exposed H9c2 cells (Fig. 4J). Additionally, cinnamaldehyde and allopurinol both at 30 μM attenuated ox-LDL-induced ROS over-production, TXNIP over-expression and CD36 up-regulation in H9c2 cells (Figs S1 and 2). Furthermore, cinnamaldehyde (20, 30 or 40 μM) and allopurinol (30 μM) blocked fructose-induced ROS over-production, TXNIP over-expression, NADPH oxidase and XOD hyperactivity in SSO-pretreated H9c2 cells (Fig. 6A–D). Moreover, they reduced fructose-induced NADPH oxidase and XOD hyperactivity (Fig. 5D,E), but not ROS overproduction and TXNIP over-expression in NAC-pretreated H9c2 cells (Fig. 5B,C). In *CD36* siRNA-transfected H9c2 cells, cinnamaldehyde (20, 30 or 40 μM) and allopurinol (30 μM) also reduced fructose-induced ROS overproduction and TXNIP over-expression (Fig. 6E,F). These data further indicate that cinnamaldehyde and allopurinol may reduce ROS to suppress myocardial cell CD36 under fructose induction.

### Cinnamaldehyde and allopurinol suppress CD36-mediated NLRP3 inflammasome activation in fructose-induced cardiac fibrosis

**Figure 7:**
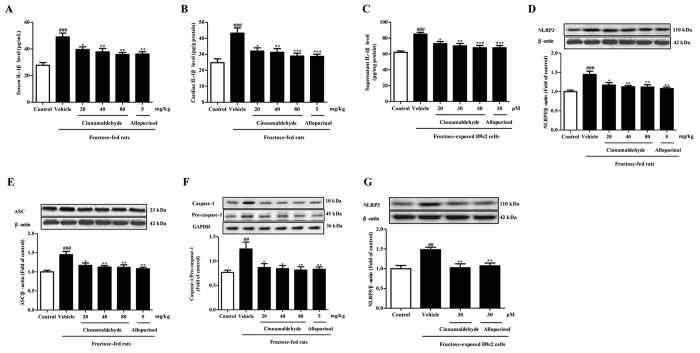
Cinnamaldehyde and allopurinol suppress fructose-induced NLRP3 inflammasome and IL-1β secretion in the heart of fructose-fed rats and fructose-exposed H9c2 cells. IL-1β levels in rat serum (**A**), rat heart (**B**) and H9c2 cells (**C**) were assayed (n = 10), respectively. Rat cardiac protein levels of NLRP3 (**D**), ASC (**E**) and Caspase-1 (**F**), as well as cellular protein levels of NLRP3 (**G**) were determined (n = 7). Relative protein levels of NLRP3 and ASC were assayed and normalized to β-actin, respectively. Relative protein levels of Caspase-1 were normalized to pro-caspase-1. Data are expressed as the mean ± SEM. ^##^*P* < 0.01, ^###^*P* < 0.001 *vs* normal animal control group or normal cell control group; *P < 0.05, **P < 0.01, ***P < 0.001 *vs* fructose-vehicle animal group or fructose-vehicle cell group.

Fructose-fed rats displayed systemic inflammation with high serum IL-1β levels (Fig. 7A). Increased IL-1β levels were also observed in the heart of fructose-fed rats and supernatant of fructose-exposed H9c2 cells (Fig. 7B,C). Simultaneously, cardiac NLRP3, ASC and mature caspase-1 protein levels were up-regulated in this animal model (Fig. 7D–F). Meanwhile, 1 mM fructose increased NLRP3 protein levels in H9c2 cells (Fig. 7G). These observations demonstrate fructose-induced NLRP3 inflammasome activation in myocardial cells.

**Figure 8:**
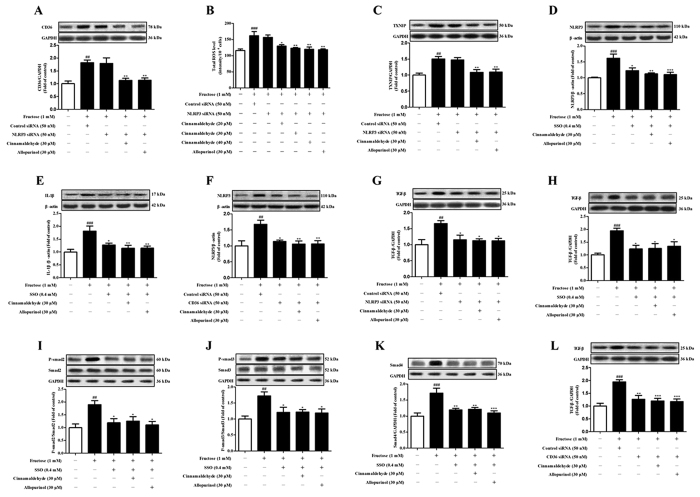
Effects of cinnamaldehyde and allopurinol on fructose-induced change of CD36, ROS, or TXNIP, NLRP3, IL-1β, TGF-β, p-Smad2/3 and Smad4 in SSO-pretreated, and *CD36* or *NLRP3* siRNA-transfected H9c2 cells. Cellular CD36 protein levels (**A**), ROS (**B**) and TXNIP protein levels (**C**) were determined in *NLRP3* siRNA-transfected H9c2 cells co-incubated with fructose, cinnamaldehyde and allopurinol (n = 7), respectively. Cellular protein levels of NLRP3 (**D**) and IL-1β (**E**) were determined in SSO-pretreated H9c2 cells co-incubated with fructose, cinnamaldehyde and allopurinol (n = 7), respectively. Cellular NLRP3 protein levels were determined in *CD36* siRNA-transfected H9c2 cells co-incubated with fructose, cinnamaldehyde and allopurinol (n = 7) (**F**). Cellular TGF-β protein levels were determined in *NLRP3* siRNA-transfected H9c2 cells co-incubated with fructose, cinnamaldehyde and allopurinol (n = 7) (**G**). Cellular protein levels of TGF-β (**H**), p-Smad2/3 (**I**,**J**) and Smad4 (**K**) were determined in SSO-pretreatedH9c2 cells co-incubated with fructose, cinnamaldehyde and allopurinol (n = 7), respectively. Cellular TGF-β protein levels (**L**) were determined in *CD36* siRNA-transfected H9c2 cells co-incubated with fructose, cinnamaldehyde and allopurinol (n = 7). The relative protein levels of CD36, TXNIP, NLRP3, IL-1β, TGF-β and Smad4 were normalized to GAPDH or β-actin, respectively. Relative protein levels of p-Smad2/3 were normalized to Smad2/3, respectively (n = 7). Data were expressed as the mean ± SEM. ^##^*P* < 0.01, ^###^*P* < 0.001 *vs* normal cell control group; *P < 0.05, **P < 0.01, ***P < 0.001 *vs* fructose-vehicle cell group, or fructose-vehicle + inhibitor/siRNA control cell group.

Ox-LDL level is closely link to cardiac structural and functional damage with inflammation response^6^. In cultured macrophage and valvular myofibroblasts, 10–50 μg/mL ox-LDL promotes proinflammatory cytokine expression^6^,^48^. In H9c2 cells treated with 25 and 50 μg/mL ox-LDL, NLRP3 and IL-1β protein levels were also increased, which were attenuated by SSO (Fig. S3), indicating that CD36 may be associated with NLRP3 inflammasome activation in myocardial cells under fructose induction. To address this, *NLRP3* siRNA-transfected H9c2 cells were co-incubated with 1 mM fructose for 24 h. *NLRP3* siRNA failed to alter CD36 up-regulation, ROS overproduction and TXNIP over-expression in fructose-exposed H9c2 cells (Fig. 8A–C). In fact, CD36-specific inhibitor SSO or *CD36* siRNA blocked fructose-induced change of NLRP3 and/or IL-1β in H9c2 cells (Fig. 8D–F). On the other hand, *NLRP3* siRNA attenuated fructose-induced TGF-β change in fructose-exposed H9c2 cells (Fig. 8G). In H9c2 cells, SSO blocked fructose-induced change of TGF-β, p-Smad2/3 and Smad4 (Fig. 8H–K), meanwhile, *CD36* siRNA abolished fructose-induced TGF-β up-regulation (Fig. 8L). These data demonstrate that CD36 may mediate NLRP3 inflammasome activation in fructose-induced cardiac fibrosis.

**Figure 9:**
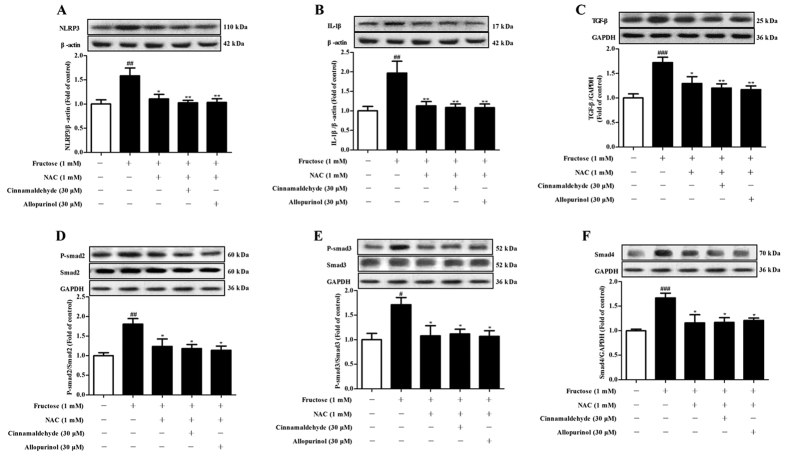
Effects of cinnamaldehyde and allopurinol on fructose-induced change of NLRP3, IL-1β, TGF-β, p-Smad2/3 and Smad4 in NAC-pretreated H9c2 cells. Cellular protein levels of NLRP3 (**A**), IL-1β (**B**), TGF-β (**C**), p-Smad2/3 (**D**,**E**) and Smad4 (**F**) were determined in NAC-pretreated H9c2 cells co-incubated with fructose, cinnamaldehyde and allopurinol, respectively. The relative protein levels of NLRP3, IL-1β, TGF-β and Smad4 were normalized to GAPDH or β-actin, respectively. Relative protein levels of p-Smad2/3 were normalized to Smad2/3, respectively (n = 7). Data were expressed as the mean ± SEM. ^#^*P* < 0.05, ^##^*P* < 0.01, ^###^*P* < 0.001 *vs* normal cell control group; *P < 0.05, **P < 0.01 *vs* fructose-vehicle cell group, or fructose-vehicle + NAC control cell group.

More importantly, ROS-specific inhibitor NAC prevented fructose-induced alteration of NLRP3 and IL-1β, as well as TGF-β, p-Smad2/3 and Smad4 in H9c2 cells (Fig. 9A–F). Cinnamaldehyde (20, 30 and 40 μM) and allopurinol (30 μM) reduced IL-1β secretion in fructose- or ox-LDL-exposed H9c2 cells (Fig. 7C, Fig. S3). Cinnamaldehyde and allopurinol both at 30 μM down-regulated NLRP3 protein levels in these cell models (Figs 7G and 3S). These data were consistent with the reduction of cardiac NLRP3 inflammasome activation (Fig. 7D–F) and IL-1β production (Fig. 7B) in animal model. Furthermore, cinnamaldehyde and allopurinol both at 30 μM reduced fructose-induced up-regulation of CD36 protein levels in *NLRP3* siRNA-transfected H9c2 cells (Fig. 8A). ROS overproduction and TXNIP over-expression were attenuated by cinnamaldehyde (20, 30 or 40 μM) and allopurinol (30 μM) (Fig. 8B–C). These results suggest that cinnamaldehyde and allopurinol may suppress cardiac oxidative stress and ROS to block CD36-mediated NLRP3 inflammasome activation in fructose-induced myocardial cell inflammation.

### Cinnamaldehyde and allopurinol inhibit CD36-mediated TLR4/6-IRAK4/1 signaling to suppress fructose-induced cardiac NLRP3 inflammasome activation

**Figure 10:**
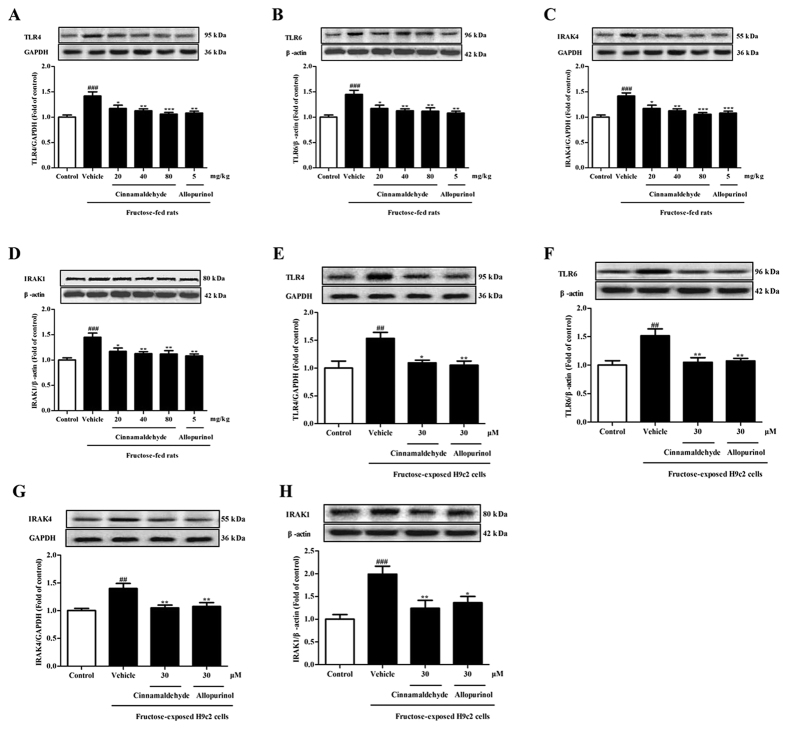
Cinnamaldehyde and allopurinol down-regulate TLR4, TLR6, IRAK4 and IRAK1 protein levels in the heart of fructose-fed rats and fructose-exposed H9c2 cells. Protein levels of rat cardiac TLR4 (**A**), TLR6 (**B**), IRAK4 (**C**) and IRAK1 (**D**), as well as cellular TLR4 (**E**), TLR6 (**F**), IRAK4 (**G**) and IRAK1 (**H**) were assayed, and relative protein levels were normalized to GAPDH or β-actin (n = 7), respectively. Data are expressed as the mean ± SEM. ^##^*P* < 0.01, ^###^*P* < 0.001 *vs* normal animal control group or normal cell control group; *P < 0.05, **P < 0.01, ***P < 0.001 *vs* fructose-vehicle animal group or fructose-vehicle cell group.

**Figure 11:**
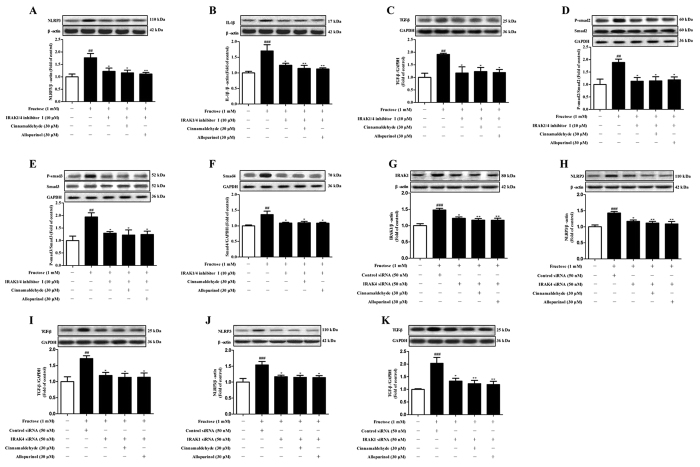
Effects of cinnamaldehyde and allopurinol on fructose-induced alteration of NLRP3 inflammasome and TGF-β/Smads signaling in IRAK1/4 inhibitor I-pretreated, and *IRAK4* or *IRAK1* siRNA-transfected H9c2 cells. Protein levels of NLRP3 (**A**), IL-1β (**B**), TGF-β (**C**), p-Smad2/3 (**D**,**E**), Smad4 (**F**) were assayed in IRAK1/4 inhibitor I-pretreated H9c2 cells co-incubated with fructose, cinnamaldehyde and allopurinol (n = 7), respectively. Protein levels of IRAK1 (**G**), NLRP3 (**H**), TGF-β (**I**) were assayed in *IRAK4* siRNA-transfected H9c2 cells co-incubated with fructose, cinnamaldehyde and allopurinol (n = 7), respectively. Protein levels of NLRP3 (**J**) and TGF-β (**K**) were assayed in *IRAK1* siRNA-transfected H9c2 cells co-incubated with fructose, cinnamaldehyde and allopurinol (n = 7), respectively. Relative protein levels of NLRP3, IL-1β, TGF-β and Smad4 were normalized to GAPDH or β-actin, respectively. Relative protein levels of p-Smad2/3 were normalized to Smad2/3, respectively (n = 7). Data are expressed as the mean ± SEM. ^##^*P* < 0.01, ^###^*P* < 0.001 *vs* normal cell control group; *P < 0.05, **P < 0.01, ***P < 0.001 *vs* fructose-vehicle cell group, or fructose-vehicle + inhibitor I/siRNA control cell group, respectively.

**Figure 12:**
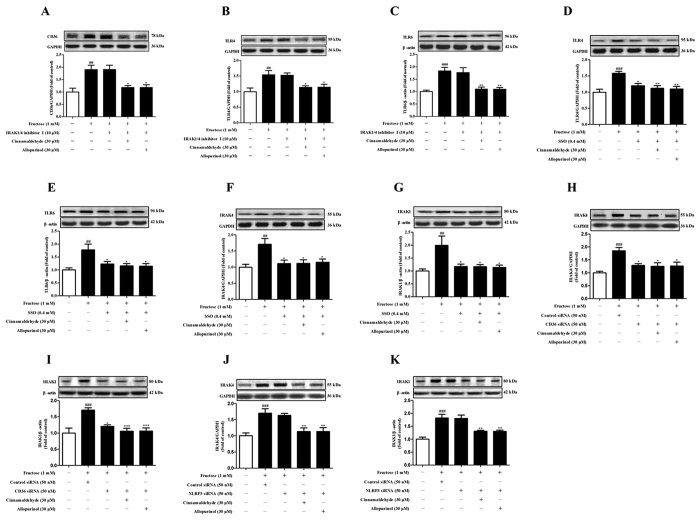
Effect of cinnamaldehyde and allopurinol on fructose-induced change of CD36, TLR4, TLR6, IRAK4 and IRAK1 protein levels in SSO-pretreated or *CD36* siRNA-transfected or IRAK1/4 inhibitor I-pretreated or *NLRP3* siRNA-transfected H9c2 cells. Protein levels of cellular CD36 (**A**), TLR4 (**B**) and TLR6 (**C**) were assayed in IRAK1/4 inhibitor I-pretreated H9c2 cells co-incubated with fructose, cinnamaldehyde and allopurinol (n = 7), respectively. Protein levels of cellular TLR4 (**D**), TLR6 (**E**), IRAK4 (**F**) and IRAK1 (**G**) were assayed in SSO- pretreated H9c2 cells co-incubated with fructose, cinnamaldehyde and allopurinol (n = 7), respectively. Protein levels of cellular IRAK4 (**H**) and IRAK1 (**I**) were assayed in *CD36* siRNA-transfected H9c2 cells co-incubated with fructose, cinnamaldehyde and allopurinol (n = 7), respectively. Protein levels of cellular IRAK4 (**J**) and IRAK1 (**K**) were assayed in *NLRP3* siRNA-transfected H9c2 cells co-incubated with fructose, cinnamaldehyde and allopurinol (n = 7), respectively. The relative protein levels were normalized to GAPDH or β-actin. Data are expressed as the mean ± SEM. ^##^*P* < 0.01, ^###^*P* < 0.001 *vs* normal cell control group; *P < 0.05, **P < 0.01, ***P < 0.001 *vs* fructose-vehicle cell group, or fructose-vehicle + inhibitor /siRNA control cell group, respectively.

NLRP3 inflammasome activation requires consecutive binding of IRAK4 and IRAK1 to TLR4/6 in macrophages of mice exposed with Listeria monocytogenes^28^,^29^. In this study, TLR4, TLR6, IRAK4 and IRAK1 protein levels were up-regulated in the heart of fructose-fed rats and fructose-exposed H9c2 cells (Fig. 10A–H). To investigate the role of IRAK4/1 in linking TLR4/6 signaling to NLRP3 inflammasome, H9c2 cells were pretreated with IRAK1/4 inhibitor I (10 μM) or transfected with *IRAK4* or *IRAK1* siRNA, and then co-incubated with 1 mM fructose for 24 h. IRAK1/4 inhibitor I reduced NLRP3 and IL-1β, as well as TGF-β, p-Smad2/3 and Smad4 in fructose-exposed H9c2 cells (Fig. 11A–F). However, this inhibitor failed to alter fructose-induced change of cellular ROS production, TXNIP over-expression, NADPH oxidase and XOD hyperactivity (Fig. 6G–J), as well as CD36 and TLR4/6 protein levels (Fig. 12A–C) in H9c2 cells. *IRAK4* siRNA suppressed IRAK1, NLRP3 and TGF-β over-expression (Fig. 11G–I), but not CD36 and ROS overproduction (Fig. 6K,L) in fructose-exposed H9c2 cells. *IRAK1* siRNA attenuated fructose-induced elevation of NLRP3 and TGF-β protein levels in H9c2 cells (Fig. 11J,K), while alteration of ROS, CD36 and IRAK4 remained unchanged (Fig. 6M–O).

Next, CD36-specific inhibitor SSO blocked fructose-induced change of TLR4, TLR6, IRAK4 and IRAK1 in H9c2 cells (Fig. 12D–G). *CD36* siRNA blocked fructose-induced change of IRAK4 and IRAK1 in H9c2 cells (Fig. 12H,I). Furthermore, *NLRP3* siRNA failed to affect fructose-induced change of IRAK4 and IRAK1 in H9c2 cells (Fig. 12J,K). These results suggest that CD36-mediated TLR4/6-IRAK4/1 signaling may participate in NLRP3 inflammasome activation in fructose-induced myocardial cell inflammation.

**Figure 13:**
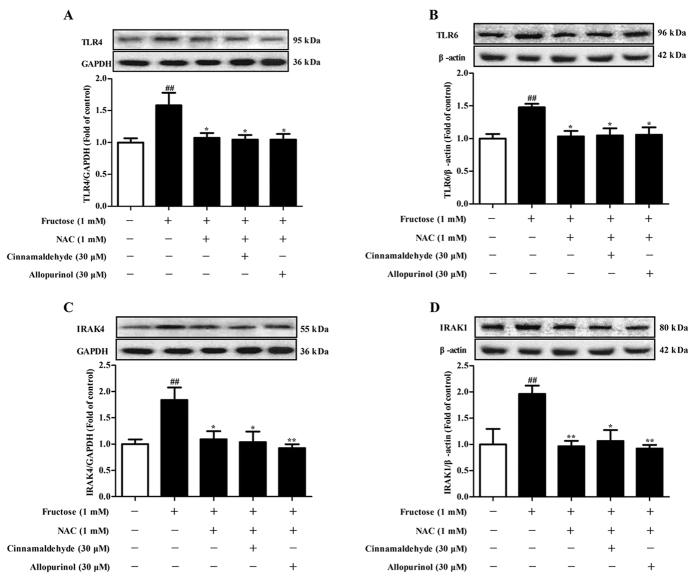
Effect of cinnamaldehyde and allopurinol on fructose-induced change of TLR4, TLR6, IRAK4 and IRAK1 protein levels in NAC-pretreated H9c2 cells. Protein levels of cellular TLR4 (**A**), TLR6 (**B**), IRAK4 (**C**) and IRAK1 (**D**) were assayed in NAC-pretreated H9c2 cells (n = 7), respectively. The relative protein levels were normalized to GAPDH or β-actin. Data are expressed as the mean ± SEM. ^##^*P* < 0.01 *vs* normal cell control group; *P < 0.05, **P < 0.01 *vs* fructose-vehicle cell group, or fructose-vehicle + NAC control cell group, respectively.

ROS-specific inhibitor NAC prevented fructose-induced alteration of TLR4/6-IRAK4/1 signaling in H9c2 cells (Fig. 13A–D). Cinnamaldehyde and allopurinol at all tested doses were found to reduce TLR4, TLR6, IRAK4 and IRAK1 protein levels in the heart of fructose-fed rats and fructose-exposed H9c2 cells (Fig. 10A–H). In IRAK1/4 inhibitor I-pretreated H9c2 cells, cinnamaldehyde (20, 30 or 40 μM) attenuated fructose-induced ROS overproduction and TXNIP over-expression (Fig. 6G,H), NADPH oxidase and XOD hyperactivity (Fig. 6I,J), as well as CD36, TLR4 and TLR6 up-regulation (Fig. 12A–C). In *IRAK4* siRNA-transfected H9c2 cells, cinnamaldehyde (20, 30 or 40 μM) reduced fructose-induced ROS overproduction and CD36 up-regulation (Fig. 6K,L). In *IRAK1* siRNA-transfected H9c2 cells, cinnamaldehyde (20, 30 or 40 μM) attenuated fructose-induced ROS overproduction, as well as CD36 and IRAK4 up-regulation (Fig. 6M–O). Allopurinol (30 μM) also had similar effects in these cells (Figs 6G–O and 12A–C). Additionally, 30 μM cinnamaldehyde and allopurinol decreased fructose-induced elevation of IRAK4 and IRAK1 protein levels in *NLRP3* siRNA-transfected H9c2 cells (Fig. 12J,K). These results suggest that cinnamaldehyde and allopurinol may reduce cardiac oxidative stress and ROS to block CD36-mediated TLR4/6-IRAK4/1 signaling and then to suppress NLRP3 inflammasome in fructose-induced myocardial cell inflammation.

## Discussion

This study for the first time demonstrated that fructose induction increased cardiac oxidative stress and ROS to up-regulate CD36, and subsequently provoked NLRP3 inflammasome in a TLR4/6-IRAK4/1-dependent manner, promoting cardiac inflammation and fibrosis in metabolic syndrome of rats with high serum ox-LDL levels. Furthermore, cinnamaldehyde and allopurinol reduced oxidative stress and ROS to down-regulate CD36, and then mediated TLR4/6-IRAK4/1 signaling to suppress NLRP3 inflammasome activation in fructose-fed rats. This study revealed that anti-oxidants cinnamaldehyde and allopurinol protected against fructose-induced cardiac inflammation and fibrosis.

Ox-LDL is a common risk factor for cardiovascular diseases^49^. CD36 as a signaling molecule binds ox-LDL and functions ox-LDL uptake. Of note, clinical study shows that high serum ox-LDL level remarkably correlates with total fructose intake in children with non-alcoholic fatty liver disease at baseline, and it is significantly reduced after low-fructose diet education for six months^7^. Patients with CD36 deficiency may be susceptible to myocardial damage with abnormal myocardial long-chain fatty acid metabolism^50^. TXNIP up-regulation is observed in cardiomyocyte impairment of diabetic mice^15^, and closely correlates with oxidative stress initiated by CD36 in ceramide-induced pancreatic β-cell dysfunction^17^. In this study, cardiac CD36 protein levels were increased in fructose-induced metabolic syndrome of rats, being consistent with elevation of serum ox-LDL levels under fructose induction. Furthermore, cardiac accumulation of ROS, TXNIP, H_2_O_2_, O_2_^·−^, ·OH and MDA were observed in fructose-fed rats with cardiac NADPH oxidase and XOD hyperactivity. Anti-oxidant enzymes SOD and CAT, as well as GSH are known to scavenge ROS. In this animal model, heart SOD and CAT activity, and GSH/GSSG ratio were significantly decreased. In fact, ox-LDL or fructose could induce ROS overproduction, TXNIP over-expression and CD36 up-regulation in H9c2 cells, which were attenuated by ROS scavenger NAC. Moreover, SSO significantly blocked ox-LDL or fructose-induced CD36 over-expression, but failed to affect fructose-induced ROS overproduction, TXNIP over-expression, NADPH oxidase and XOD hyperactivity in H9c2 cells. In *CD36* siRNA-transfected H9c2 cells, ROS overproduction and TXNIP over-expression induced by fructose were not reduced. These observations indicate that fructose-induced cardiac oxidative stress and ROS may closely promote myocardial cell CD36 up-regulation in high ox-LDL-associated cardiovascular diseases.

High ox-LDL level is closely linked to cardiac structural and functional injury in response to inflammation^6^. CD36-deficient macrophages reduce IL-1β release by activating NLRP3 inflammasome^13^. Under fructose or ox-LDL induction, NLRP3 inflammasome was activated with IL-1β secretion in rat heart and/or H9c2 cells. More importantly, CD36-specific inhibitor SSO blocked these changes in H9c2 cells. Smad2/3 phosphorylation and fibrosis in response to TGF-β1 is attenuated in Nlrp3^−/−^ H293T cells^51^. NLRP3-deficient cardiac fibroblasts impair differentiation and R-Smad activation in response to TGF-β^21^. Subsequently, the present study found that fructose induced high TGF-β, p-Smad2/3 and Smad4 levels in rat heart and H9c2 cells. SSO also blocked fructose-induced elevation of TGF-β, p-Smad2/3 and Smad4 in H9c2 cells. *CD36* siRNA suppressed fructose-induced change of NLRP3 and TGF-β in H9c2 cells. Additionally, *NLRP3* siRNA attenuated TGF-β change but not ROS overproduction in this cell model. These observations indicate that ROS-triggered CD36 may activate NLRP3 inflammasome to promote cardiac inflammation and fibrosis in fructose-induced heart injury.

TLRs are a key link between innate immunity and cardiovascular diseases^23^. Ox-LDL up-regulates TLR4 expression in peripheral blood monocytes from healthy subjects^24^. CD36 causes the assembly of a heterodimer of TLR4 and TLR6^24^. TLR signaling molecule IRAK1 seems to regulate NLRP3 inflammasome assembly and activation^28^. Deletion of IRAK1 or IRAK4 causes defective inflammasome activation by *Listeria monocytogenes*^28^,^29^. In this study, TLR4 and TLR6 were found to be up-regulated in the heart of fructose-fed rats and fructose-exposed H9c2 cells. Accordantly, IRAK4 and IRAK1 were also increased in these animal and cell models. Of note, IRAK1/4 inhibitor I blocked fructose-induced NLRP3 activation, as well as TGF-β, p-Smad2/3 and Smad4 up-regulation in H9c2 cells, without change of CD36 and TLR4/6. *IRAK4* or *IRAK1* siRNA abolished fructose-induced alteration of NLRP3 and TGF-β, but not ROS and CD36 in H9c2 cells. In fact, CD36 specific inhibitor attenuated fructose-induced elevation of TLR4/6, IRAK4/1, NLRP3, IL-1β, TGF-β, p-Smad2/3 and Smad4 in H9c2 cells. *CD36* siRNA also reduced IRAK4, IRAK1, NLRP3 and TGF-β in fructose-exposed H9c2 cells. These data indicate that CD36-mediated TLR4/6-IRAK4/1 signaling may participate in NLRP3 inflammasome activation in fructose-induced cardiac inflammation and fibrosis. Of note, ROS inhibitor retarded the activation of CD36-mediated TLR4/6-IRAK4/1 signaling, as well as NLRP3 and TGF-β/Smads signaling in fructose-exposed H9c2 cells. These observations suggest that fructose may increase ROS and CD36 to provoke NLPR3 inflammasome activation and TGF-β/Smads signaling in a TLR4/6-IRAK4/1-dependent manner, thus promoting cardiac inflammation and fibrosis.

Cinnamaldehyde improves metabolic disorders in rodents, decreases ROS production and IL-1β secretion in lipopolysaccharide-stimulated murine J774A.1 macrophages, suppresses plasma TLR4 expression in myocardium of viral myocarditis mice, and alleviates ischemic myocardial injury of rats, exhibiting its anti-oxidative and anti-inflammatory property^32^,^34^,^35^,^36^. Allopurinol retards NLRP3 inflammasome activation in fructose-induced metabolic syndrome of rats^39^,^40^ and ameliorates high-fat and high-fructose diet-induced oxidative stress, inflammation and cardiomyocyte hypertrophy in mice^41^. In the present study, cinnamaldehyde dose-dependently significantly alleviated oxidative stress, ROS over-production and TXNIP over-expression in fructose-fed rat heart and H9c2 cells. It could down-regulate CD36 protein levels in these animal and cell models. Allopurinol also reduced oxidative stress and CD36 in fructose-fed rat heart and H9c2 cells. In ox-LDL-exposed H9c2 cells, cinnamaldehyde and allopurinol reduced over-production of ROS, and over-expression of TXNIP, CD36, NLRP3 and IL-1β. Furthermore, cinnamaldehyde and allopurinol remarkably abolished fructose-induced ROS overproduction and TXNIP over-expression, but not NADPH oxidase and XOD hyperactivity in NAC-pretreated H9c2 cells. In *CD36* siRNA-transfected or CD36 specific inhibitor-pretreated H9c2 cells, cinnamaldehyde and allopurinol reduced fructose-induced ROS overproduction, TXNIP over-expression, NADPH oxidase and XOD hyperactivity. These results indicate that cinnamaldehyde and allopurinol may reduce oxidative stress and ROS, and subsequently decrease CD36 expression in myocardial cells under fructose induction.

NLRP3 inflammasome inhibitor and NLRP3 mutant procedure alleviate cardiac injury in mice^20^. In this study, cinnamaldehyde and allopurinol inhibited NLRP3 inflammasome activation to reduce IL-1β release and TGF-β/Smads signaling in animal and cell models. Of note, they only reduced fructose-induced ROS and CD36 in *NLRP3* siRNA-transfected H9c2 cells. These results suggest that cinnamaldehyde and allopurinol may reduce cardiac oxidative stress and ROS to block CD36-mediated NLRP3 inflammasome activation in fructose-induced myocardial cell inflammation and fibrosis.

Consistently, cinnamaldehyde and allopurinol down-regulated TLR4/6 and IRAK4/1 in the heart of fructose-fed rats and fructose-exposed H9c2 cells. Indeed, they prevented fructose-induced ROS overproduction, TXNIP over-expression, NADPH oxidase and XOD hyperactivity, CD36 and TLR4/6 up-regulation in IRAK1/4 inhibitor I-pretreated H9c2 cells. Cinnamaldehyde and allopurinol also reduced fructose-induced ROS, TXNIP and CD36 in *IRAK4* or *IRAK1* siRNA-transfected H9c2 cells. Our findings suggest that the blockage of oxidative stress and ROS to suppress NLRP3 inflammasome activation by cinnamaldehyde and allopurinol may be dependent on the suppression of CD36-mediated TLR4/6-IRAK4/1 signaling in fructose-induced cardiac inflammation and fibrosis.

Fructose consumption is closely associated with the development of cardiovascular disease, due to its induction of XOD hyperactivity-mediated intracellular and serum uric acid^41^,^42^. Allopurinol suppresses XOD and protects heart against ROS, inflammation and fibrosis in high fat and high fructose diet-fed rats^41^. It also alleviates low grade inflammation and cardiac ischemia in fructose-induced hyperuricemia of rats^42^. Allopurinol lowers serum uric acid levels, resulting in the improvement of blood pressure in fructose-induced metabolic syndrome in patients^52^, and reduces free radical production in type 1 diabetes patients with a potentially higher oxidant buildup as a result of increased cardiovascular risk^53^,^54^. It also reduces serum SBP and ox-LDL in cardiac surgery patients with hyperuricemia, and alters expression of inflammatory markers in patients with acute ischemic stroke^55^,^56^. Thus, allopurinal is clinically suggested as cardioprotectant^57^,^58^,^59^. Cinnamaldehyde is a major and important compound from cinnamon. It exhibits potent XOD inhibitory activity to reduce serum uric acid levels in hyperuricemic mice^60^. Cinnamaldehyde can reduce serum TG, TC and LDL-c levels in patients with type 2 diabetes^32^, improve glucose tolerance in high fat diet-induced obese mice^61^ and protect against cerebral ischaemia injury in mice by reducing IL-1β production^62^. Therefore, the attenuation of oxidative stress and ROS to suppress CD36-mediated TLR4/6-IRAK4/1 signaling and NLRP3 inflammasome activation by cinnamaldehyde and allopurinol may be a promising therapeutic strategy for the treatment of metabolic syndrome-associated heart disease. High fructose triggers sophisticated systemic and cardiac oxidative stress, which may be a risk factor in heart injury of metabolic syndrome. More investigations are needed to determine the role of fructose-induced oxidative stress in heart injury, and explore in anti-oxidant mechanisms of cinnamaldehyde and allopurinol in alleviating cardiac inflammation and fibrosis under fructose induction.

In conclusion, this study demonstrates that cinnamaldehyde and allopurinol reduce oxidative stress and ROS to alleviate heart injury in fructose-induced metabolic syndrome of rats. Furthermore, they suppress NLPR3 inflammasome activation to reduce IL-1β and TGF-β/Smads signaling via CD36-mediated TLR4/6-IRAK4/1 signaling in animal and cell models, exhibiting the alleviation of fructose-induced cardiac inflammation and fibrosis. These results suggest that the inclusion of cinnamon in the diet or the treatment of allopurinol in subjects with fructose-induced metabolic syndrome may reduce risk factors associated with heart diseases.

## Materials and Methods

### Animals

Male Sprague-Dawley rats aged from 8 to 10 weeks (200–220 g) were purchased from the Experimental Animal Centre of Nanjing Medical University (Nanjing, China) (Production license: SCXK2008-0004). They were housed at 22 ± 2 °C under a relative humidity of 55 ± 5% and a normal 12-h light/dark cycle with the lights on at 6:00 a.m. Rats were given a standard chow and water *ad libitum* for the study and one week for acclimatization before the experiment. Each rat was given 100 mL drinking water or drinking water containing 10% fructose (wt/vol) (Jiakangyuan Science and Technology Co., Ltd., Beijing, China) and standard chow for 10 weeks. After 5-week fructose feeding, rats were randomized into five subgroups (n = 15/group), receiving drinking water, 20, 40 and 80 mg/kg cinnamaldehyde (95% purity) and 5 mg/kg allopurinol (98% purity), (Sigma, St. Louis, MO, USA) for additional 5 weeks, respectively. All tested samples were given orally once daily at 2:00–3:00 p.m..

It is reported that cinnamaldehyde alleviates hyperlipidemia in C57BLKS/J db/db mice at 20 mg/kg^31^, reduces oxidative stress in myocardial tissue in a rat model of ischemic myocardial injury at 22.5, 45 and 90 mg/kg^36^, and protects against cerebral ischaemia injury of mice at 25, 50 and 75 mg/kg^62^. Our previous study showed that allopurinol at 5 mg/kg reduced oxidative stress and NLRP3 inflammasome activation in the liver and kidney of fructose-fed rats^39^,^63^. Accordingly, based on our preliminary work and these reports, the doses of 20, 40 and 80 mg/kg cinnamaldehyde, as well as 5 mg/kg allopurinol were used in the present study.

Additionally, 15 rats were remained on regular chow for 10 weeks to serve as normal control. Animal body weight was detected weekly. Animal welfare and experimental procedures were carried out in accordance with the recommendations in the guidelines of the Ministry of Science and Technology of China (2006) and the related ethnical regulations of Nanjing University [SYXK (SU) 2009-0017]. All experimental protocol involving animals were approved by the Institutional Animal Care and Use Committee of Nanjing University. All efforts were made to minimize animal suffering and to reduce the number of animals used.

### Body weight and 24-h food intake

At the end of 10 weeks, rat body weight and 24-h food intake were detected in a metabolic cage as previously described, respectively^43^.

### SBP

SBP was measured by the tail-cuff system (Softron BP-98A; Softron, Tokyo, Japan) at the end of 10 weeks and the rats were conscious. Data were averaged for six-seven consecutive measurements.

### OGTT and ITT

During the last week of feeding period, OGTT and ITT were performed as described before^43^. Briefly, rats were orally administered with glucose (1.5 g/kg) or intraperitoneally injected with insulin (0.8 IU/kg) (Sinopharm Chemical Reagent Co., Ltd., Shanghai, China). Tail-vein blood samples were collected at 0, 30, 60, 90 and 120 min after glucose or insulin treatment, and then centrifuged (4000× *g*, 4 °C) for 10 min to get serum for glucose assay.

### Blood and tissue samples collection

After OGTT and ITT, all animals were allowed 3 days to recover wounds. Then, animals were anesthetized intraperitoneally using 50 mg/kg sodium pentobarbital and decapitated at 9:00–10:00 a.m. after a 16-h fast. Blood samples were centrifuged for 10 min to get the serum stored at −80 °C for biochemical assays. Heart tissue samples were rapidly dissected on ice. Parts of them were immediately fixed for oil red O or Masson trichrome staining, while others were stored at −80 °C for biochemical and Western blot analysis.

### Oil red O and Masson trichrome staining analysis

Rat heart tissues were fixed for one day at room temperature in Carnoy’s fixative (ethanol: chloroform: acetic acid = 6:3:1) and preserved in 70% ethanol. Cardiac biopsies were dehydrated with a graded series of alcohol and embedded in paraffin. Specimens were cut in 4 μm-thick sections on a rotary microtome and mounted on 3-aminopropyltriethoxysilane-coated glass slides. Each section was washed by distilled water and then stained with oil red O reagent (Jiancheng Biotechnology Co., Ltd., Nanjing, China) for 5–10 min. After washed with 60% isopropyl alcohol, the sections were deparaffinized in xylene, rehydrated in decreasing concentrations of alcohol in water, and re-stained with Masson trichrome reagent (Google Biological Technology Co., Ltd., Wuhan, China), respectively. The slides were mounted with neutral balsam.

### Cell culture

H9c2 cells were supported by Shanghai fuxiang Biotechnology Co., Ltd (Shanghai, China). H9c2 cells were maintained in DMEM (4.5 g/L glucose), supplemented with 10% FBS (Wisent Technology, St-Bruno, QC, Canada) in a humidified 5% CO_2_ atmosphere at 37 °C. During experiments, H9c2 cells were plated in 6-well plates (2 mL/well, 2.5 × 10^5^ cells/mL), 12-well plates (1 mL/well, 2.5 × 10^5^ cells/mL) or 96-well plates (200 μL/well, 5 × 10^4^ cells/mL) for 12 h, respectively. Then H9c2 cells were grown to confluence and made quiescent by incubation in serum-free DMEM for 12 h. These cells were maintained in DMEM (containing 10% FBS) and exposed with 0.1% DMSO alone (normal control), 1 mM fructose (fructose-vehicle), 1 mM fructose co-incubated with 20, 30 and 40 μM cinnamaldehyde or 30 μM allopurinol for 24 h, respectively.

To explore the molecular mechanisms, H9c2 cells were grown to confluence and made quiescent by incubation in serum-free DMEM for 12 h. H9c2 cells were pretreated with ROS-specific inhibitor NAC (1 mM, Amresco, Solon, USA) for 1 h, or CD36-specific inhibitor SSO (0.4 mM, Santa Cruz Biotechnology Co., Ltd., USA) for 0.5 h, respectively, and then were co-incubated with 25 and 50 μg/mL ox-LDL (Yiyuan Biotechnologies, Guangzhou, China) for other 24 h. Meanwhile, H9c2 cells were co-incubated with 25 and 50 μg/mL ox-LDL, in the presence or absence of cinnamaldehyde (30 μM) or allopurinol (30 μM) for 24 h.

Furthermore, H9c2 cells were pretreated with SSO (0.4 mM) for 0.5 h, or NAC (1 mM) for 1 h or IRAK1/4 inhibitor I (10 μM, MedChem Express, Shanghai, China) for 1 h, respectively, and then were co-incubated with 1 mM fructose in the presence or absence of cinnamaldehyde (20, 30 and 40 μM) or allopurinol (30 μM) for other 24 h. SSO and IRAK1/4 inhibitor I were observed to reduce protein levels of CD36 (Fig. S4, Fig. 12A), IRAK4 (Fig. 12F, Fig. S5) and IRAK1 (Fig. 12G, Fig. S6) in fructose-exposed H9c2 cells, respectively.

*CD36*, *NLRP3*, *IRAK4* and *IRAK1* siRNA, as well as the respective negative control were synthesized by GenePharma (Shanghai, China), respectively. The primers used were listed in Supplementary Table S1. Transfection of *CD36*, *IRAK4*, *IRAK1 or NLRP3* siRNA (50 nM), as well as respective negative control in H9c2 cells were performed for 6 h using Lipofectamine 2000 (Invitrogen, Carlsbad, CA, USA) according to the manufacturer’s instructions, respectively. These cells were then incubated with fresh culture for other 18 h before co-incubated with fructose, cinnamaldehyde or allopurinol. The efficiency of *CD36*, *IRAK4*, *IRAK1* or *NLRP3* siRNA (Figs S7–10) in H9c2 cells at 24 h-transfection was detected by quantitative real-time PCR (qRT-PCR, Bio-Rad CFX96 Real-Time PCR Detection System), respectively. After transfection, these cells were incubated with 1 mM fructose in the presence or absence of cinnamaldehyde (20, 30 and 40 μM) or allopurinol (30 μM) for other 24 h.

It is reported that 10–40 μM cinnamaldehyde inhibits IL-1β secretion in lipopolysaccharide or lipoteichoic acid stimulated-murine J774A.1 macrophages^34^. Cinnamaldehyde at 40 μM prevents lipid accumulation in ethanol-induced 3T3-L1 preadipocyte cell line^64^. In our previous studies^39^,^63^, 1–5 μM allopurinol restores fructose-induced ROS over-prodiction, TXNIP over-expression and NLRP3 inflammasome activation in primary rat hepatocytes at 5 μM, or mouse podocytes at 100 μM. Accordingly, based on these observations and our preliminary experiment, dosages of cinnamaldehyde (20, 30 and 40 μM), as well as of allopurinol (30 μM) were selected for the cell experiments.

Cinnamaldehyde, allopurinol, SSO or IRAK1/4 inhibitor I dissolved in DMSO, NAC dissolved in ultrapure water at the respective stock concentrations, were directly added to cell culture medium. The final concentration of DMSO in culture medium was maintained at 0.1%. The selected concentrations and incubation time of these reagents were referred to preliminary experiments and other reports^65^,^66^,^67^,^68^,^69^. Cell culture supernatants were collected. Cell lysates were obtained by cell lysis buffer, and total cellular proteins were extracted, respectively. These samples were stored at −80 °C before biochemical and Western blot analysis.

### RNA isolation and qRT-PCR analysis

Total RNA was isolated from H9c2 cells using Trizol reagent (Invitrogen) according to the manufacturer’s instructions, respectively. The reverse transcription reaction of mRNAs has been previously published^62^. The primers used were listed in Supplementary Table S1. The reverse transcription reaction products were amplified by qRT-PCR with iTaq^TM^ Universal SYBR^®^ Green Supermix (Bio-Rad) and respective primers. Specificity of the amplification was confirmed using a melting curve analysis. Data were collected and recorded by CFX Manager Software (Bio-Rad), and expressed as a function of threshold cycle (Ct). The samples for qRT-PCR analysis were evaluated using a single predominant peak as a quality control. Relative expressions of target genes were determined by the Ct (2^−ΔΔCt^) method. mRNAs were normalized to glyceraldehyde-3-phosphate dehydrogenase (GAPDH), respectively.

### Assay of TG, TC, LDL-c and ox-LDL concentrations

Lipids were extracted from rat serum, heart tissue and H9c2 cells by liquid phase extraction using chloroform/methanol (2:1) as previously described^43^. TG, TC and LDL-c levels were determined with standard diagnostic kits (Jiancheng Biotechnology Co., Ltd., Nanjing, China), respectively. Rat heart tissue samples were homogenized in lysis buffer and then centrifuged at 12000× *g* for 15 min at 4 °C. Ox-LDL concentrations in serum was determined by ELISA kit (Shanghai Lianshuo Biological Technology Co., Ltd., Shanghai, China).

### Determination of oxidative stress

Heart tissue was homogenized in PBS and centrifuged (10,000× *g*, 4 °C) for 15 min. H9c2 cells were detached from the wells by 0.25% trypsin digestion. Total ROS assay (Beyotime Institute of Biotechnology, Haimei, China) was performed with cell-containing aliquots (transferred to 96-well plate at 1 × 10^4^ cells/well), while NADPH oxidase and XOD activity assays were performed on cell lysates using a buffer (P0013J; Beyotime Biotech, Nanjing, China) or the lysis buffer provided in assay kits. XOD activity in heart tissue homogenate and/or H9c2 cell lysates was determined by standard diagnostic kit (Jiancheng Biotechnology Co., Ltd., Nanjing, China). Activity of NADPH oxidase was represented as the rate of NADPH consumption according to the previously described^70^. Standard diagnostic kits were used for the determination of O_2_^·−^, ^·^OH, SOD and MDA levels (Jiancheng Biotechnology Co., Ltd., Nanjing, China), of H_2_O_2_, GSH, GSSG and CAT levels (Beyotime Institute of Biotechnology, Haimei, China) in heart tissue homogenate, respectively.

### IL-1β assay

Rat heart tissues were homogenized in 10 wt/vol of sodium chloride on ice, and then centrifuged (10,000× *g*, 4 °C) for 15 min to collect the supernatants. IL-1β levels in serum, tissue and cell supernatant were determined by commercial ELISA kit (IBL, Minneapolis, MN, USA), respectively.

### Determination of hydroxyproline levels

Hydroxyproline levels in heart tissues were performed by standard diagnostic kit (Jiancheng Biotechnology Co., Ltd., Nanjing, China).

### Western blot analysis

Protein sample preparation and Western blot analysis were performed using standard procedures. Rat heart tissue samples homogenized in lysis buffer and cell lysates were centrifuged (3000× *g*, 4 °C) for 15 min. These supernatant samples were again centrifuged at 12,000× *g* for 20 min (4 °C).

After resolution of sample protein (equal loading for each sample) by 10% SDS-PAGE, the protein was electrophoretically transferred onto PVD membranes. The primary antibodies included: rabbit anti-CD36 (ab133625) purchased from Abcam (Cambridge, MA, USA); rabbit anti-TGF-β (#3711), rabbit anti-Smad2 (#5339), rabbit anti-Smad3 (#9523), rabbit anti-Smad4 (#9515), rabbit anti-Phospho-Smad2 (#3108), rabbit anti-Phospho-Smad3 (#9520), rabbit anti-NLRP3 (#13158), rabbit anti-ASC (#4628) and rabbit anti-IRAK4 (#4363) purchased from Cell Signaling Technology (Cambridge, USA); mouse anti-pro-IL-1β (MAB5011) and mouse anti-IL-1β (MAB5011) purchased from R&D System (Minneapolis, USA) (dilution 1:1000); rabbit anti-pro-caspase-1 (sc-514), rabbit anti-caspase-1 (sc-514) (dilution 1:300), rabbit anti-TLR6 (sc-30001), rabbit anti-TLR4 (sc-30002), rabbit anti-IRAK1 (sc-7883) (dilution 1:200), anti-TXNIP (AM20296AF-N, dilution 1:1000) purchased from ACRIS (Los Angeles, USA), and rabbit anti-GAPDH (sc-25778, dilution 1:500) purchased from Santa Cruz Biotechnology Co., Ltd. (Santa Cruz, CA, USA); rabbit anti-β-actin (#SAP1647, dilution 1:12000) purchased from Sunshine Bio-Tech Co., Ltd. (Nanjing, China).

Blots were incubated overnight at 4 °C in primary antibody in 5% milk followed by HRP-conjugated anti-rabbit IgG antibody (074-1506, dilution 1:10000, KPL) or HRP-conjugated anti-mouse IgG antibody (sc-2005, dilution 1:10000, Santa Cruz Biotechnology Co., Ltd.). Immunoreactive bands were visualized via enhanced chemiluminescence (Cell Signaling Technology) and quantified via densitometry using ImageJ (version 1.42q, National Institutes of Health).

### Statistical analysis

All data were expressed as the mean ± SEM and statistical analysis was performed using a one-way analysis of variance (ANOVA), followed by the Turkey’s Multiple Comparison Test. A value of P < 0.05 was considered statistically significant.

## Additional Information

**How to cite this article**: Kang, L.-L. *et al.* Cinnamaldehyde and allopurinol reduce fructose-induced cardiac inflammation and fibrosis by attenuating CD36-mediated TLR4/6-IRAK4/1 signaling to suppress NLRP3 inflammasome activation. *Sci. Rep.* **6**, 27460; doi: 10.1038/srep27460 (2016).

## Supplementary Material

Supplementary Information

## Figures and Tables

**Table 1:** Effects of cinnamaldehyde and allopurinol on body weight, 24-h food intake, heart weight and SBP in fructose-fed rats.

Group	Dose (mg/kg)	Body weight (g)	24-h food intake (g/kg/day)	Heart-to-body (HW/BW) (%)	SBP (mmHg)
Normal control	—	418.12 ± 35.74	34.75 ± 2.86	0.32 ± 0.02	110.50 ± 1.36
Fructose + vehicle	—	513.80 ± 25.71^###^	24.11 ± 3.69^###^	0.38 ± 0.03^###^	143.50 ± 3.99^###^
Fructose + cinnamaldehyde	20	488.07 ± 43.97	29.16 ± 2.74*	0.35 ± 0.02	127.16 ± 3.09
Fructose + cinnamaldehyde	40	475.48 ± 19.68*	28.96 ± 2.63*	0.34 ± 0.02*	123.33 ± 7.21*
Fructose + cinnamaldehyde	80	462.80 ± 22.06**	29.86 ± 4.22**	0.34 ± 0.01**	120.83 ± 2.38**
Fructose + allopurinol	5	466.97 ± 15.67**	30.23 ± 3.53**	0.33 ± 0.01**	116.66 ± 3.10***

Data are expressed as the mean ± SEM. (n = 10). ^###^*P* < 0.001 *vs* normal animal control group, *P < 0.05, **P < 0.01, ***P < 0.001 *vs* fructose-vehicle animal group.

**Table 2:** Effects of cinnamaldehyde and allopurinol on hyperlipidemia, and cardiac lipid accumulation and hydroxyproline levels in fructose-fed rats.

Group	Dose (mg/kg)	LDL-c (mg/dL)	Serum level			Cardiac level		
			ox-LDL (ng/mL)	TG (mg/dL)	TC (mg/dL)	TG (mg/g protein)	TC (mg/g protein)	Hydroxyproline (μg/g protein)
Normal control	—	22.04 ± 1.35	20.61 ± 2.79	73.70 ± 4.18	52.72 ± 2.31	5.70 ± 0.53	3.20 ± 0.37	247.45 ± 15.65
Fructose + vehicle	—	38.98 ± 2.42^###^	42.93 ± 2.34^###^	163.52 ± 9.43^###^	85.03 ± 4.67^###^	12.27 ± 0.47^###^	9.07 ± 0.33^###^	446.97 ± 33.44^###^
Fructose + cinnamaldehyde	20	29.92 ± 2.58*	32.37 ± 3.25	130.70 ± 6.39*	73.68 ± 2.29*	9.65 ± 1.04	6.15 ± 0.84	326.70 ± 31.45
Fructose + cinnamaldehyde	40	28.10 ± 1.96**	30.04 ± 1.89**	125.78 ± 8.62**	71.11 ± 1.83**	8.40 ± 0.96*	5.40 ± 0.96**	301.66 ± 13.30*
Fructose + cinnamaldehyde	80	26.90 ± 1.73***	26.81 ± 1.64***	119.53 ± 8.22**	66.61 ± 1.06***	7.90 ± 0.53**	4.90 ± 0.53**	306.86 ± 43.07*
Fructose+allopurinol	5	27.14 ± 1.43**	26.32 ± 2.99***	124.69 ± 6.27**	66.17 ± 2.37***	7.80 ± 0.96**	4.80 ± 0.96**	294.57 ± 29.38**

Data are expressed as the mean ± SEM. (n = 10). ^###^*P* < 0.001 *vs* normal animal control group, *P < 0.05, **P < 0.01, ***P < 0.001 *vs* fructose-vehicle animal group.

**Table 3:** Effects of cinnamaldehyde and allopurinol on cardiac H_2_O_2_, O_2_^·−^, ^·^OH and MDA, GSH/GSSG ratio, SOD and CAT in fructose-fed rats.

Group	Dose (mg/kg)	H_2_O_2_ level (μmol/g protein)	O_2_^·−^ level (U/g protein)	^·^OH level (U/mg protein)	MDA level (nmol/mg protein)	GSH/GSSG ratio	SOD activity (U/mg)	CAT activity (U/mg protein)
Normal control	—	16.77 ± 1.28	27.13 ± 3.17	90.44 ± 7.95	0.20 ± 0.00	4.75 ± 0.41	0.45 ± 0.01	390.31 ± 13.68
Fructose + vehicle	—	22.16 ± 0.59^###^	45.05 ± 3.15^###^	139.21 ± 7.00^###^	0.31 ± 0.01^###^	1.43 ± 0.09^###^	0.27 ± 0.02^###^	308.81 ± 11.87^###^
Fructose + cinnamaldehyde	20	18.31 ± 0.81*	31.93 ± 1.29*	100.74 ± 9.24*	0.23 ± 0.01*	2.97 ± 0.37	0.34 ± 0.03*	356.15 ± 9.00*
Fructose + cinnamaldehyde	40	17.68 ± 0.85**	31.75 ± 2.80*	98.49 ± 6.55**	0.22 ± 0.02**	3.18 ± 0.46*	0.35 ± 0.01**	360.61 ± 7.15*
Fructose + cinnamaldehyde	80	17.56 ± 0.75**	30.19 ± 3.53**	95.66 ± 8.69**	0.21 ± 0.01**	3.49 ± 0.39**	0.38 ± 0.01***	375.87 ± 9.21***
Fructose + allopurinol	5	17.54 ± 0.78**	29.13 ± 2.04**	98.41 ± 7.39**	0.20 ± 0.01***	3.42 ± 0.39**	0.41 ± 0.01***	367.34 ± 13.49**

Data are expressed as the mean ± SEM. (n = 10). ^###^*P* < 0.001 *vs* normal animal control group, *P < 0.05, **P < 0.01, ***P < 0.001 *vs* fructose-vehicle animal group.
