# Supplementary material for: Cinnamaldehyde and allopurinol reduce fructose-induced cardiac inflammation and fibrosis by attenuating CD36-mediated TLR4/6-IRAK4/1 signaling to suppress NLRP3 inflammasome activation
Source: Sci Rep. 2016 Jun 8;6:27460. doi: 10.1038/srep27460 (PMC4897702; doi:10.1038/srep27460)
Supplement: Supplementary Information [file srep27460-s1.doc]

**Cinnamaldehyde and allopurinol reduce fructose-induced cardiac inflammation and fibrosis by attenuating CD36-mediated TLR4/6-IRAK4/1 signaling to suppress NLRP3 inflammasome activation**

Lin-Lin Kang^*^, Dong-Mei Zhang^*^, Chun-Hua Ma, Jian-Hua Zhang, Ke-Ke Jia, Jia-Hui Liu, Rong Wang & Ling-Dong Kong

State Key Laboratory of Pharmaceutical Biotechnology, School of Life Science, Nanjing University, Nanjing, People’s Republic of China

Correspondence and requests for materials should be addressed to L.-D.K. (email: kongld@nju.edu.cn)

^*^ These two authors contribute to this work equally.

Supplementary Figures and Table


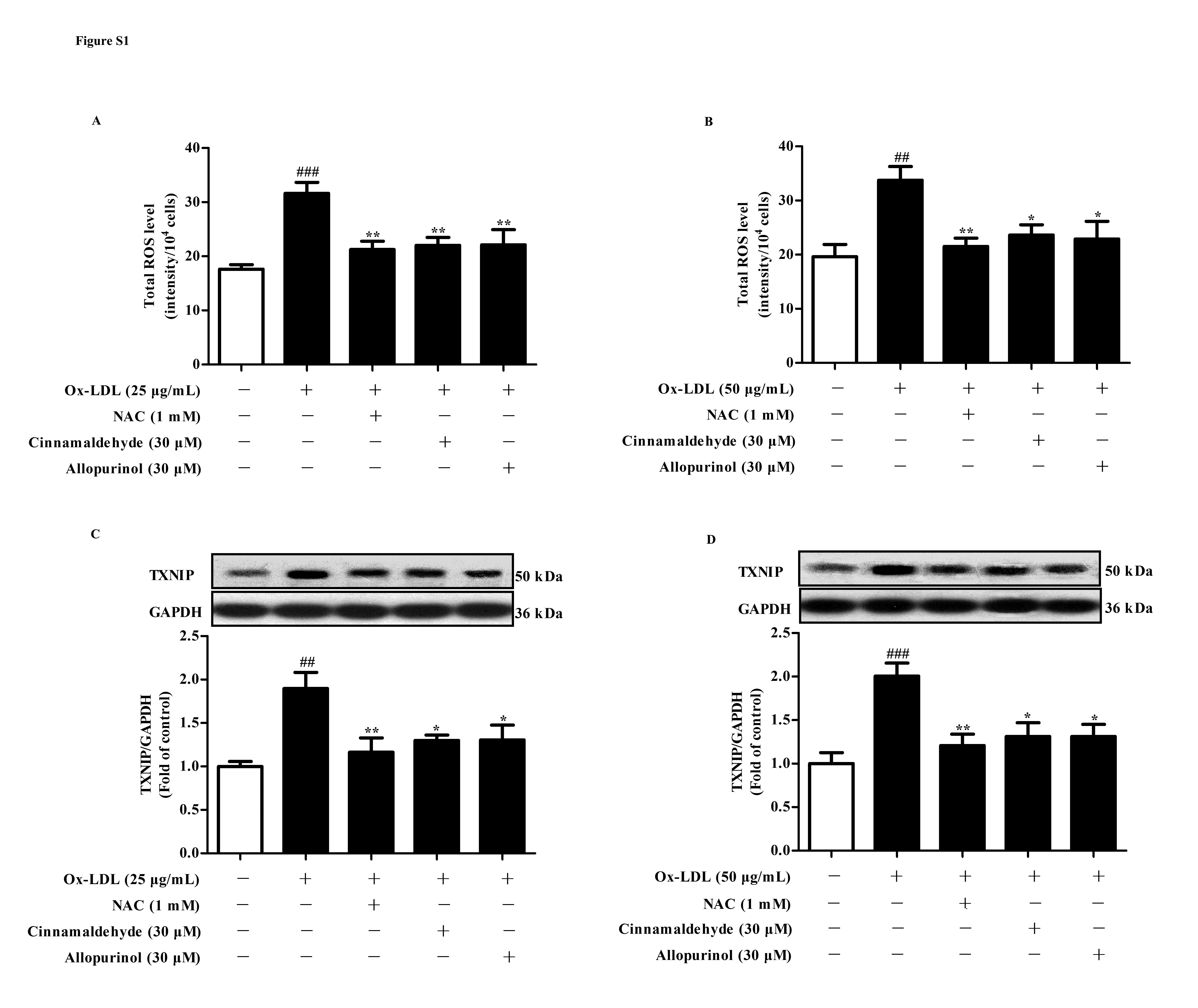


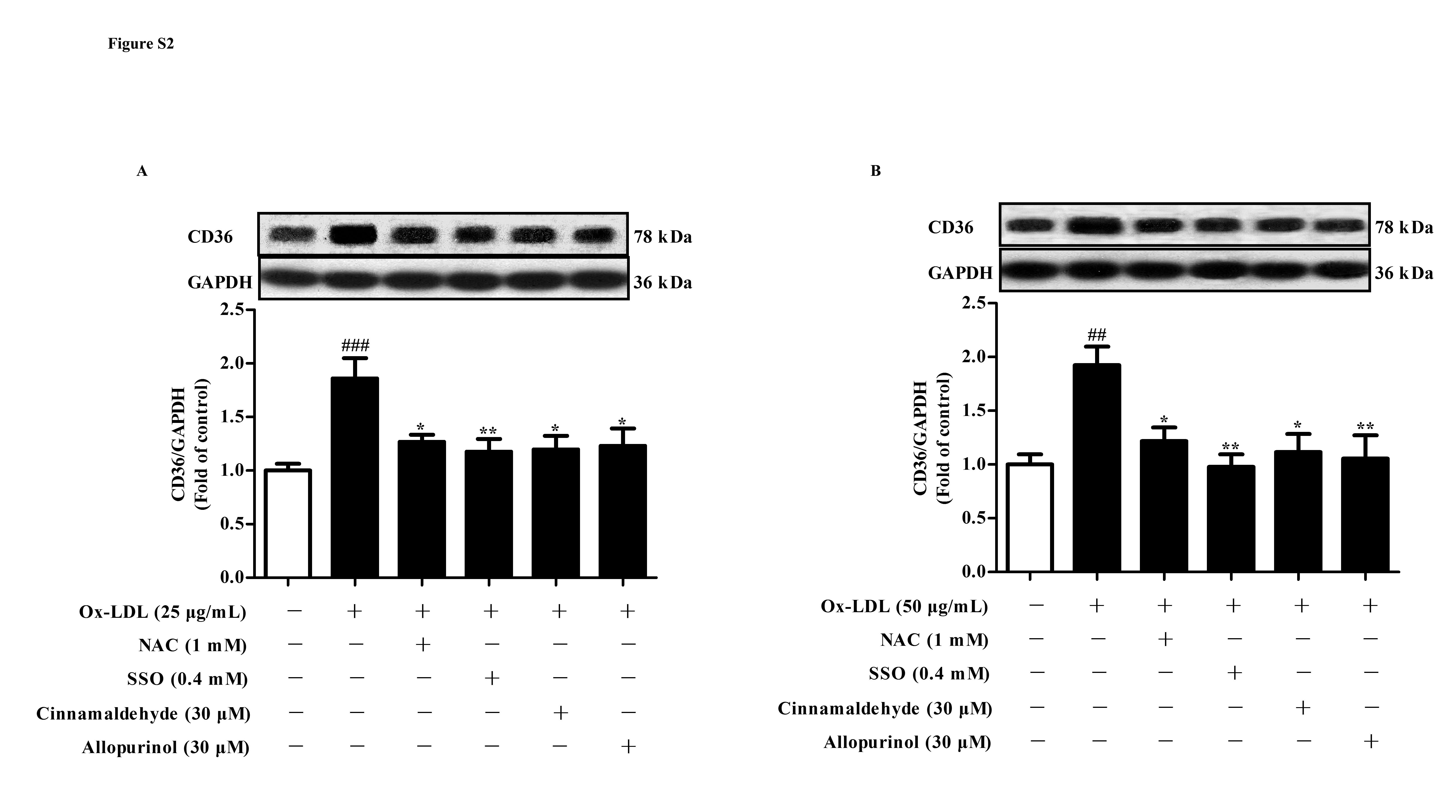


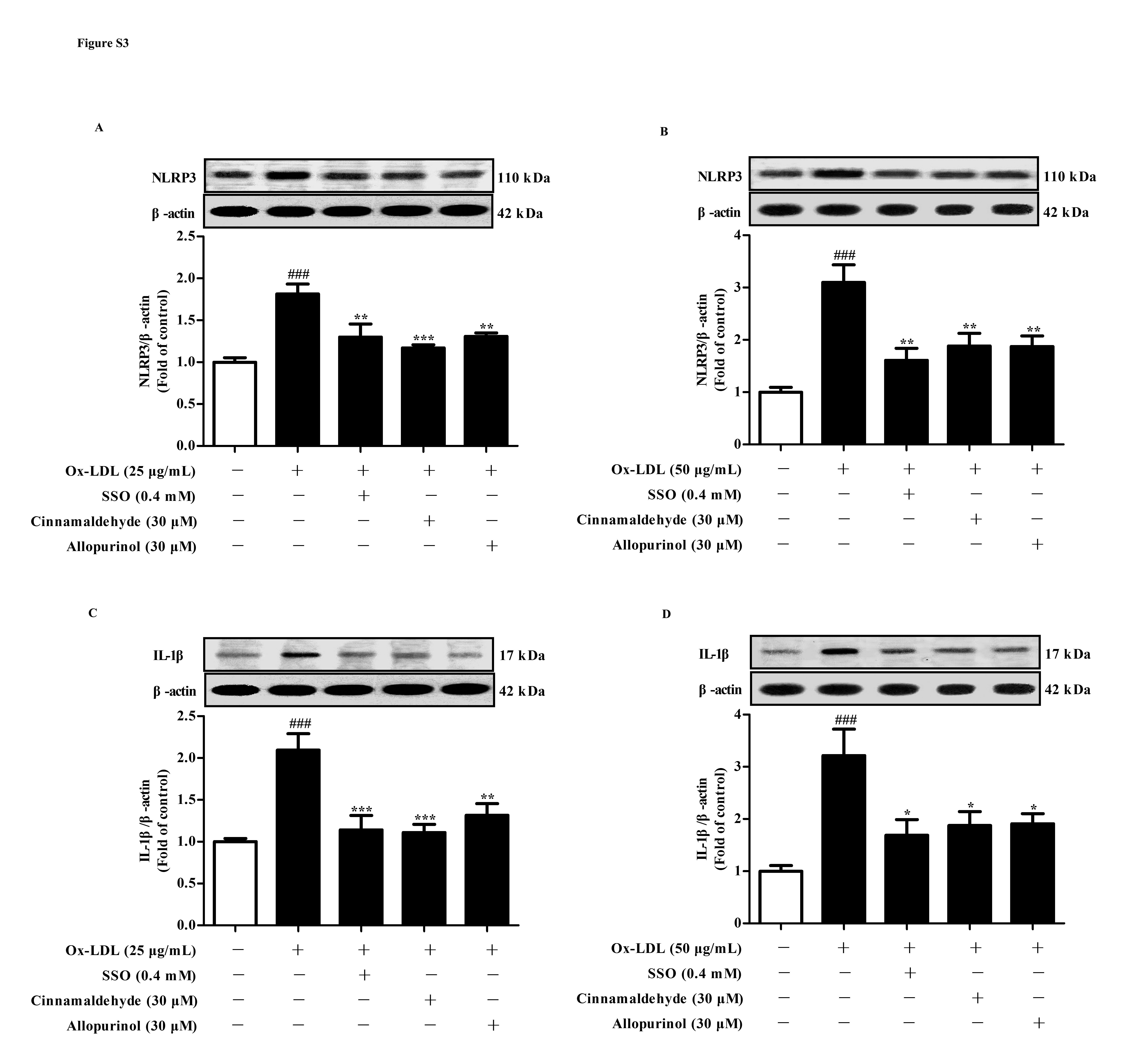


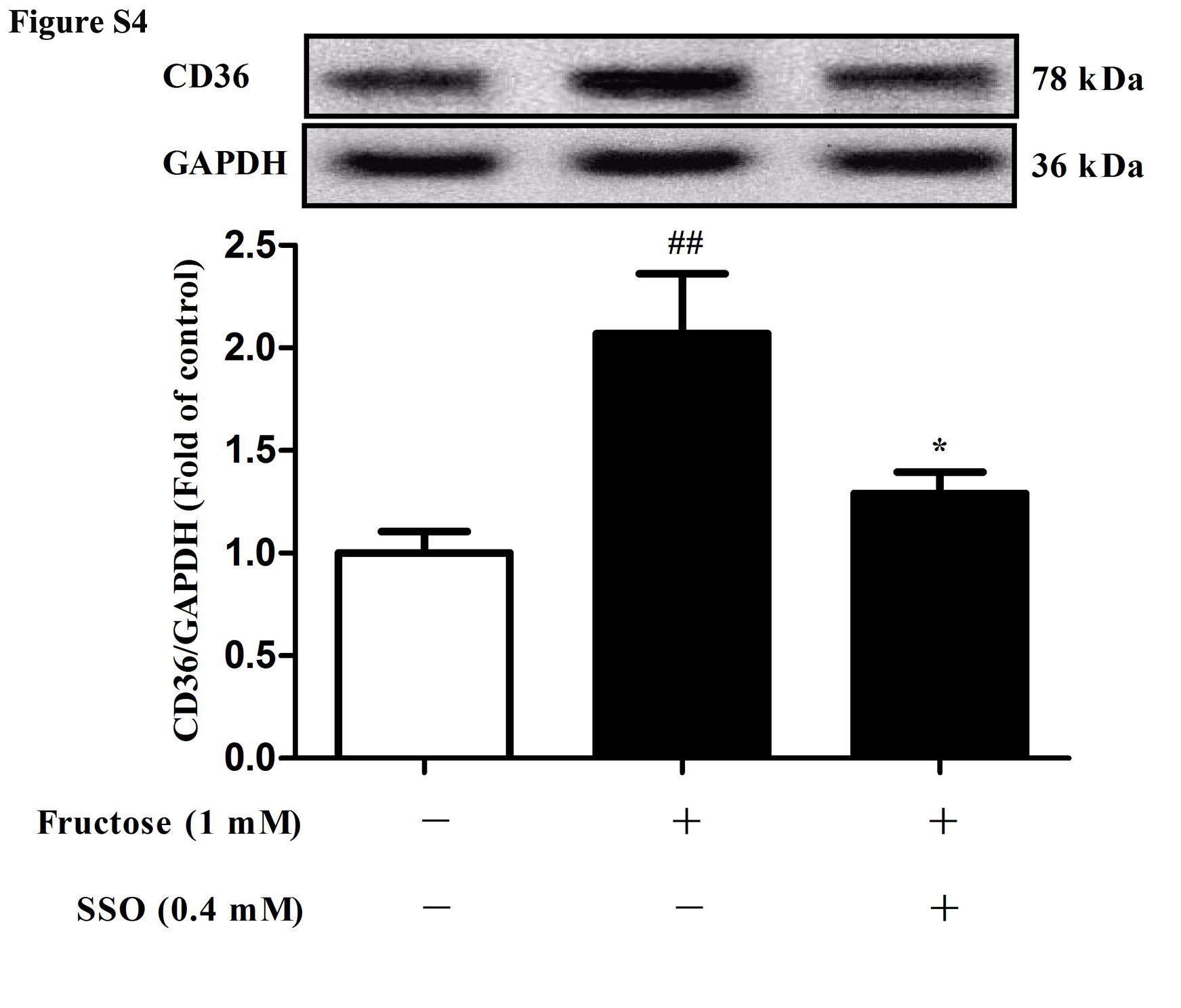


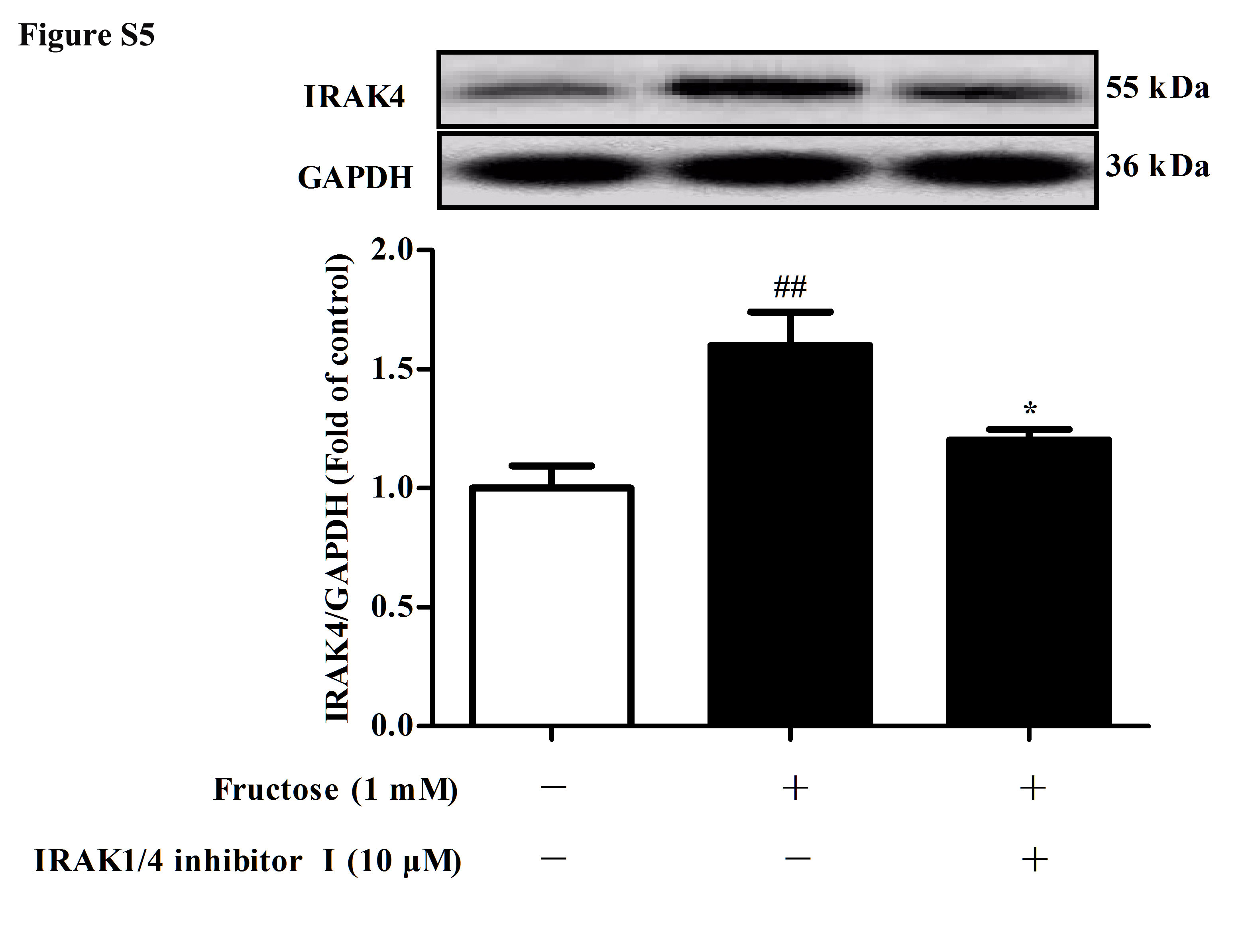


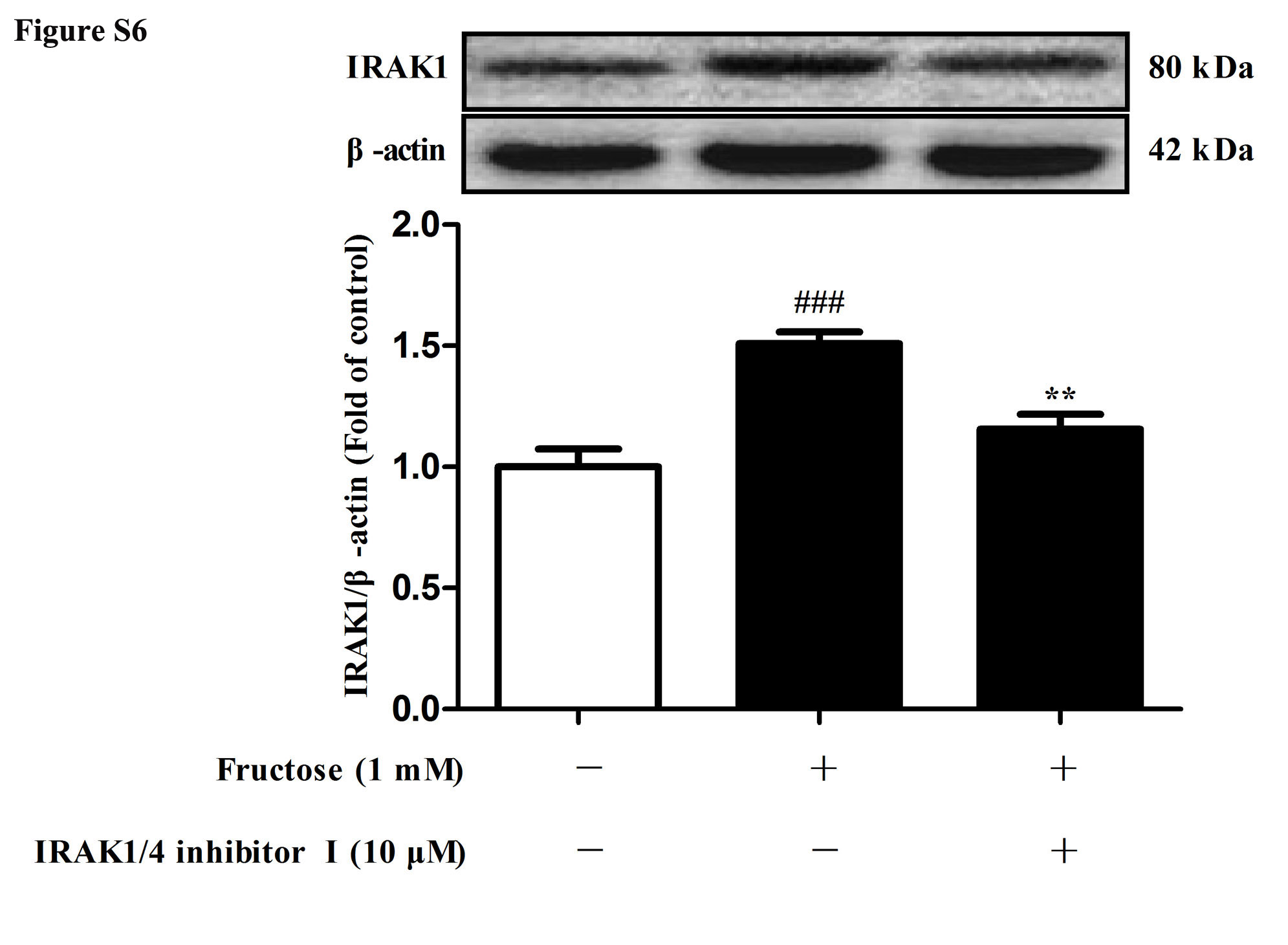


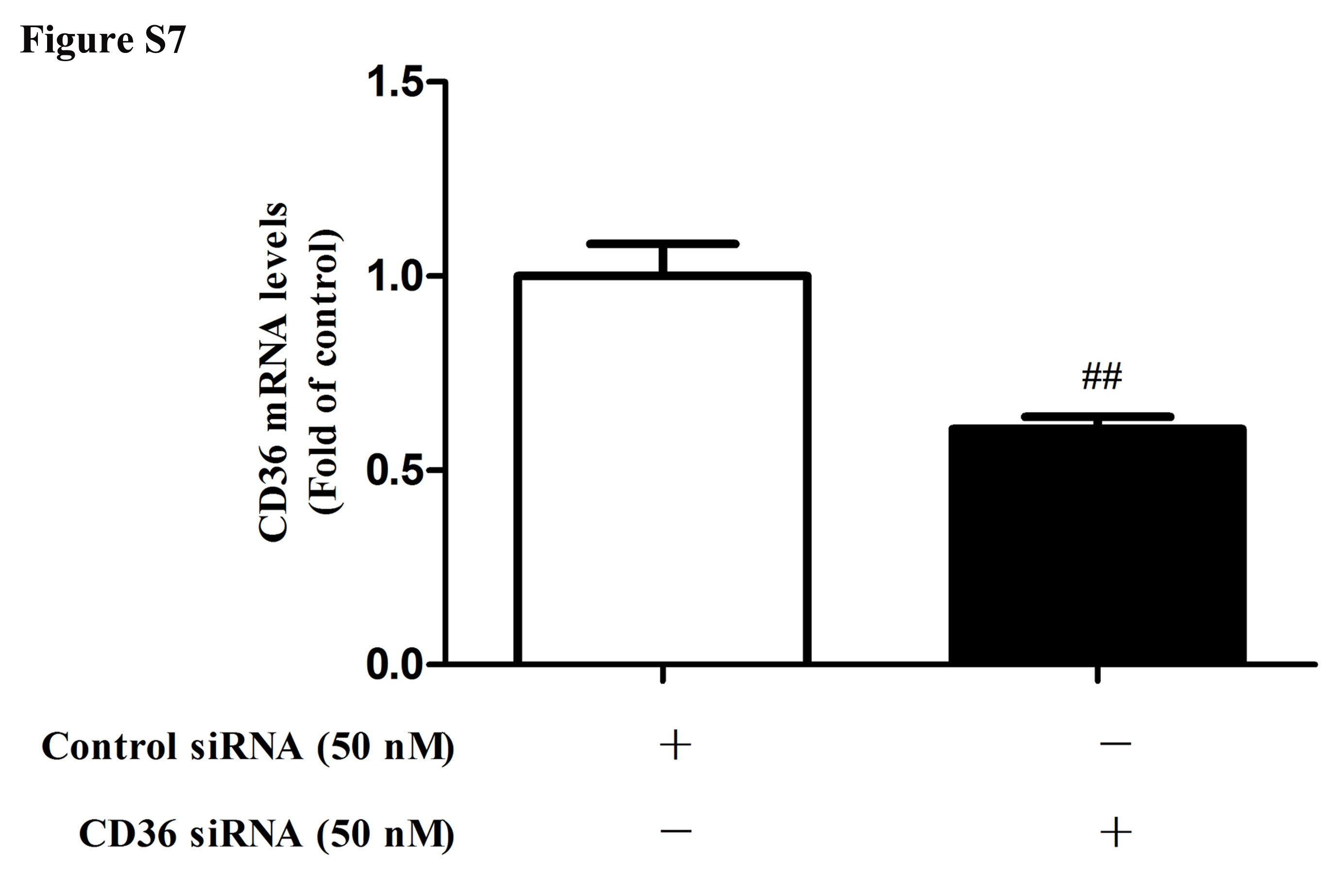


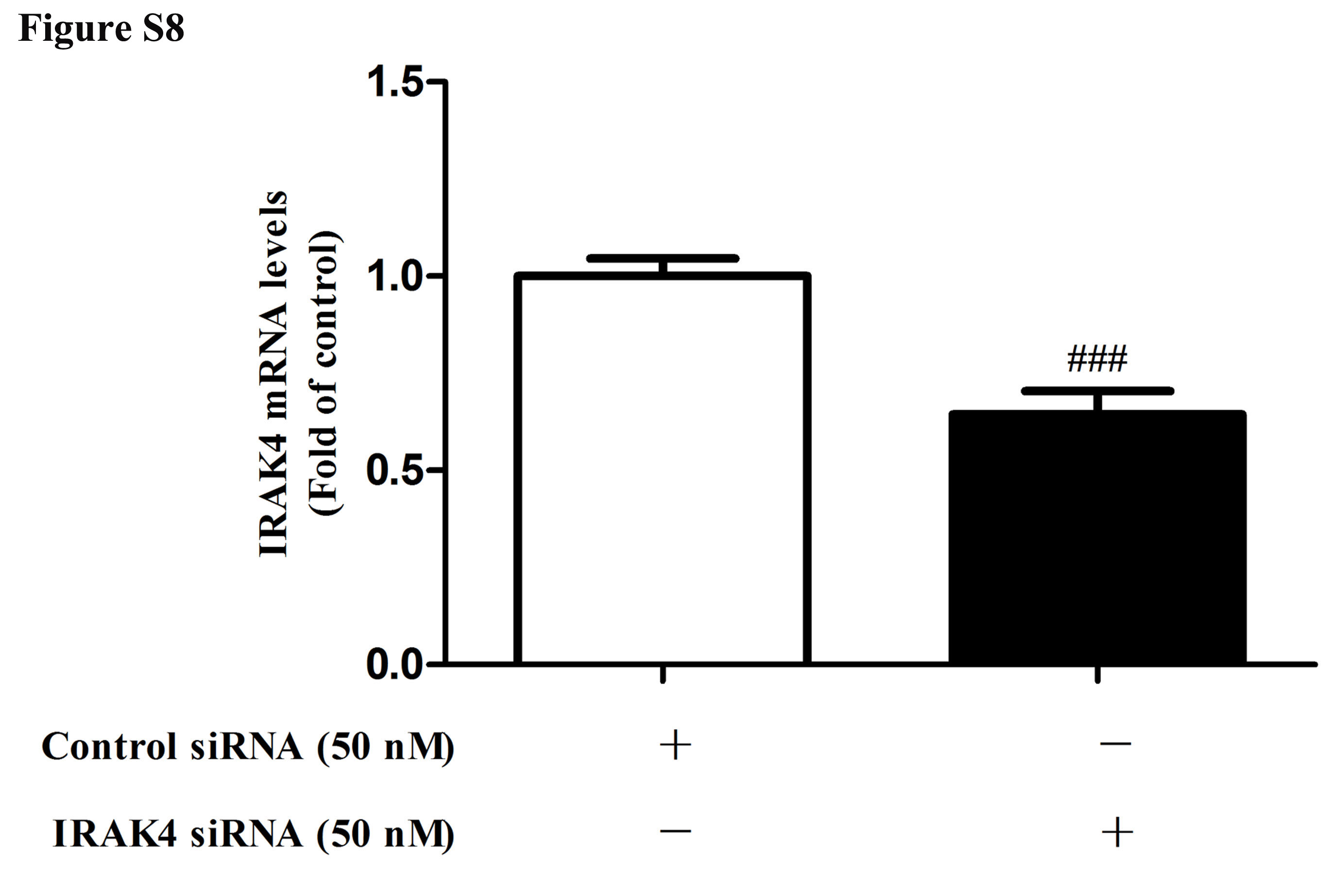


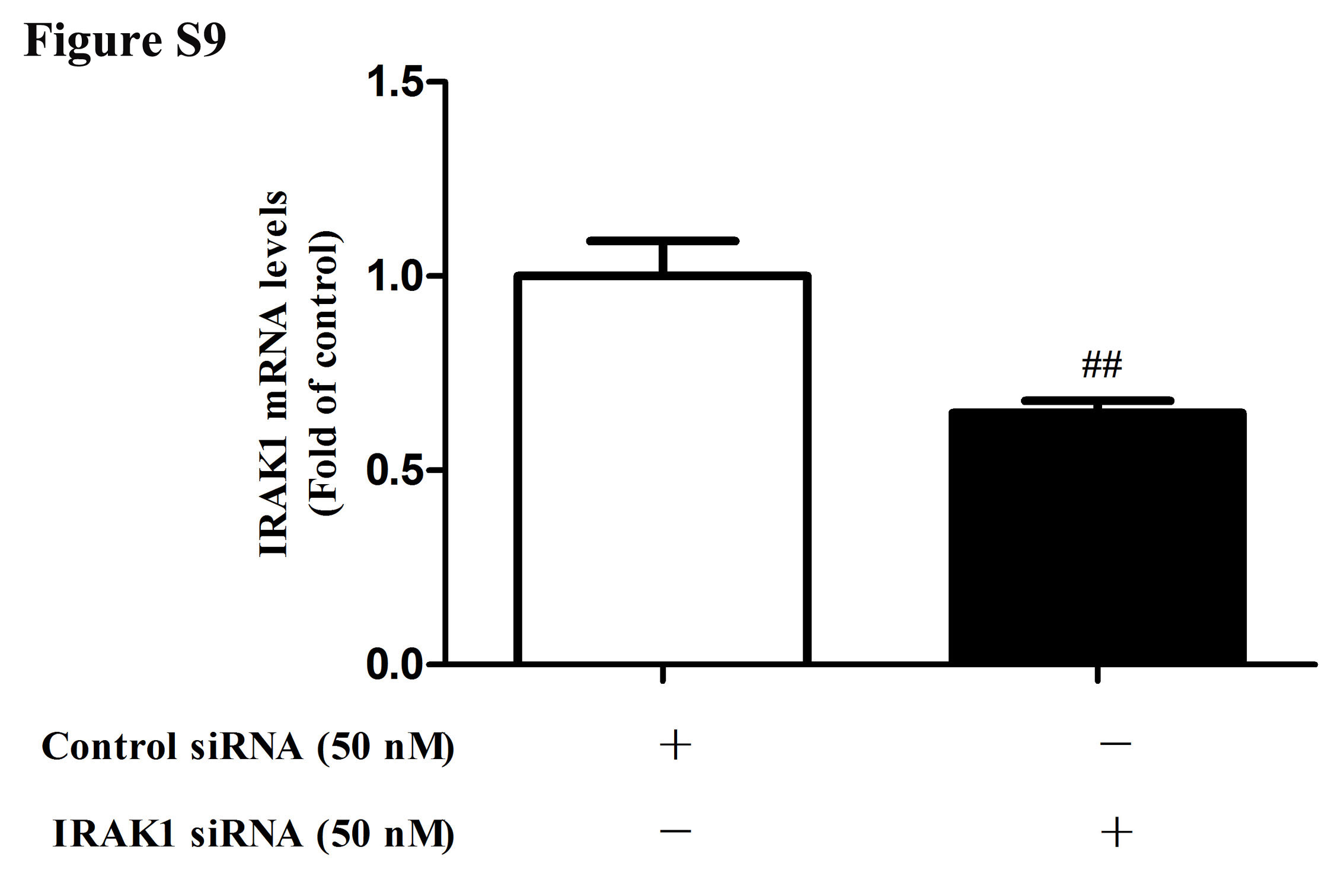


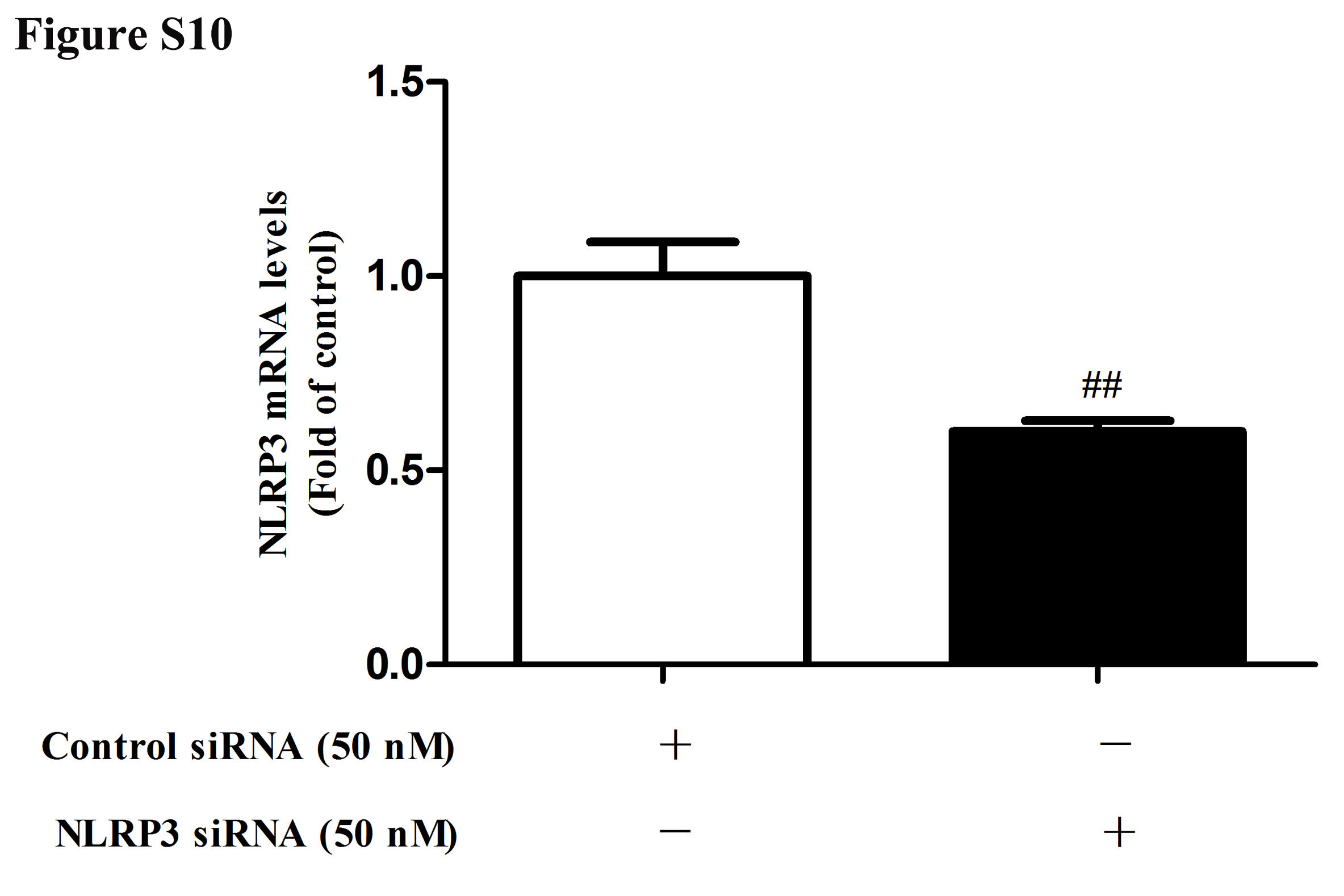


**Table S1. siRNA and primer sequences.**

| ID | Sense primer (5´→ 3´) | Antisense primer (5´→ 3´) |
| --- | --- | --- |
| CD36 | CGGTTGGAGACCTACTCATTG | GCTGCTATTCTTTGCCACTTC |
| IRAK4 | CCAAGTGTCAGCACGAAAAC | CAGTCTGTCTAGCAAGGAACCA |
| IRAK1 | TGGTAATACTGGAGACCCTTGC | AAGCATCCGTGGCTACACC |
| NLRP3 | AGAGGAGTGGATAGGTTTGCTG | TGGGTGTAGCGTCTGTTGAG |
| GAPDH | AAGAAGGTGGTGAAGCAGG | GAAGGTGGAAGAGTGGGAGT |
| CD36-siRNA | CCUUGAAGAAGGAACCAUUTT | AAUGGUUCCUUCUUCAAGGTT |
| IRAK4-siRNA | CGGGUGAUGACAGAUACAATT | UUGUAUCUGUCAUCACCCGTT |
| IRAK1-siRNA | GCUUCUACUGCCUUGUUUATT | UAAACAAGGCAGUAGAAGCTT |
| NLRP3-siRNA | GCUUCAGCCACAUGACUUUTT | AAAGUCAUGUGGCUGAAGCTT |
| Negative control | UUCUCCGAACGUGUCACGUTT | ACGUGACACGUUCGGAGAATT |

**Figure legends**

**Figure S1. NAC, cinnamaldehyde and allopurinol attenuate ox-LDL-induced ROS overproduction and TXNIP up-regulation in H9c2 cells.**

Cellular ROS levels (A-B) and TXNIP protein levels (C-D) were determined in 25 or 50 μg/mL ox-LDL-exposed H9c2 cells co-incubated with NAC, cinnamaldehyde or allopurinol (n=5-8), respectively. Data are expressed as the mean ± SEM. ^##^*P* < 0.01, ^###^*P* < 0.001 *vs* normal cell control group; ^*^*P* < 0.05, ^**^*P* < 0.01, ^***^*P* < 0.001 *vs* ox-LDL-vehicle cell group, respectively.

**Figure S2. NAC, SSO, cinnamaldehyde and allopurinol reduce ox-LDL-induced CD36 up-regulation in H9c2 cells.**

Cellular CD36 protein levels (A-B) were determined in 25 or 50 μg/mL ox-LDL-exposed H9c2 cells co-incubated with NAC, SSO, cinnamaldehyde or allopurinol (n=5), respectively. The relative protein levels were assayed and normalized to GAPDH. Data are expressed as the mean ± SEM. ^##^*P* < 0.01, ^###^*P* < 0.001 *vs* normal cell control group; ^*^*P* < 0.05, ^**^*P* < 0.01 *vs* ox-LDL-vehicle cell group, respectively.

**Figure S3. SSO, cinnamaldehyde and allopurinol down-regulate NLRP3 and IL-1β protein levels in ox-LDL-exposed H9c2 cells.**

Cellular NLRP3 (A-B) and IL-1β protein levels (C-D) were determined in 25 or 50 μg/mL ox-LDL-exposed H9c2 cells co-incubated with SSO, cinnamaldehyde or allopurinol (n=5), respectively. The relative protein levels were assayed and normalized to β-actin. Data are expressed as the mean ± SEM. ^##^*P* < 0.01, ^###^*P* < 0.001 *vs* normal cell control group; ^*^*P* < 0.05, ^**^*P* < 0.01 *vs* ox-LDL-vehicle cell group, respectively.

**Figure S4.** **CD36-specific inhibitor SSO reduces fructose-induced CD36 in H9c2 cells.** CD36 protein levels were assayed in SSO-pretreated H9c2 cells co-incubated with 1 mM fructose (n=7). The relative protein levels were normalized to GAPDH. Data are expressed as the mean ± SEM. ^###^*P* < 0.001 *vs* normal cell control group; ^*^*P* < 0.05, *vs* fructose-vehicle cell group, respectively.

**Figure S5.** **IRAK4/1 inhibitor I decreases fructose-induced IRAK4 in H9c2 cells.** IRAK4 protein levels were assayed in IRAK4/1 inhibitor I-pretreated H9c2 cells co-incubated with 1 mM fructose (n=7). The relative protein levels were normalized to GAPDH. Data are expressed as the mean ± SEM. ^###^*P* < 0.001 *vs* normal cell control group; ^*^*P* < 0.05 *vs* fructose-vehicle cell group, respectively.

**Figure S6.** **IRAK4/1 inhibitor I down-regulates IRAK1 in H9c2 cells.** IRAK1 protein levels were assayed in IRAK4/1 inhibitor I-pretreated H9c2 cells co-incubated with fructose (n=7). The relative protein levels were normalized to GAPDH. Data are expressed as the mean ± SEM. ^###^*P* < 0.001 *vs* normal cell control group; ^**^*P* < 0.01 *vs* fructose-vehicle cell group, respectively.

**Figure S7. The transfection efficiency of CD36 gene silencing in H9c2 cells.** CD36 mRNA levels were assayed in *CD36* siRNA- and control siRNA-transfected H9c2 cells (n=7), respectively. The relative mRNA levels were normalized to GAPDH. Data are expressed as the mean ± SEM. ^##^*P* < 0.01 *vs* siRNA control cell group.

**Figure S8.** **The transfection efficiency of IRAK4 gene silencing in H9c2 cells.** IRAK4 mRNA levels were assayed in *IRAK4* siRNA- and control siRNA-transfected H9c2 cells (n=7), respectively. The relative mRNA levels were normalized to GAPDH. Data are expressed as the mean ± SEM.  ^###^*P* < 0.001 *vs* siRNA control cell group.

**Figure S9.** **The transfection efficiency of IRAK1 gene silencing in H9c2 cells.** IRAK1 mRNA levels were assayed in *IRAK1* siRNA- and control siRNA-transfected H9c2 cells (n=7), respectively. The relative mRNA levels were normalized to GAPDH. Data are expressed as the mean ± SEM. ^##^*P* < 0.01 *vs* siRNA control cell group.

**Figure S10.** **The transfection efficiency of NLRP3 gene silencing in H9c2 cells.** NLRP3 mRNA levels were assayed in NLRP3 siRNA- and control siRNA-transfected H9c2 cells (n=7), respectively. The relative mRNA levels were normalized to GAPDH. Data are expressed as the mean ± SEM. ^##^*P* < 0.01 *vs* siRNA control cell group.
